# Ultrasensitive Photodetection in MoS_2_ Avalanche Phototransistors

**DOI:** 10.1002/advs.202102437

**Published:** 2021-08-08

**Authors:** Junseok Seo, Jin Hee Lee, Jinsu Pak, Kyungjune Cho, Jae‐Keun Kim, Jaeyoung Kim, Juntae Jang, Heebeom Ahn, Seong Chu Lim, Seungjun Chung, Keehoon Kang, Takhee Lee

**Affiliations:** ^1^ Department of Physics and Astronomy and Institute of Applied Physics Seoul National University Seoul 08826 Korea; ^2^ Department of Energy Science Sungkyunkwan University Suwon 16149 Korea; ^3^ Center for Integrated Nanostructure Physics Institute for Basic Science (IBS) Sungkyunkwan University Suwon 16149 Korea; ^4^ Soft Hybrid Materials Research Center Korea Institute of Science and Technology Seoul 02792 Korea; ^5^ Max‐Planck Institute of Microstructure Physics Halle Saale 06120 Germany; ^6^ Department of Smart Fabrication Technology Sungkyunkwan University Suwon 16149 Korea; ^7^ KHU‐KIST Department of Converging Science and Technology Kyung Hee University Seoul 02447 Korea; ^8^ Department of Materials Science and Engineering Yonsei University Seoul 03722 Korea

**Keywords:** avalanche photodetectors, electrical breakdown, field‐effect transistors, photoresponsivity, transition metal dichalcogenide

## Abstract

Recently, there have been numerous studies on utilizing surface treatments or photosensitizing layers to improve photodetectors based on 2D materials. Meanwhile, avalanche breakdown phenomenon has provided an ultimate high‐gain route toward photodetection in the form of single‐photon detectors. Here, the authors report ultrasensitive avalanche phototransistors based on monolayer MoS_2_ synthesized by chemical vapor deposition. A lower critical field for the electrical breakdown under illumination shows strong evidence for avalanche breakdown initiated by photogenerated carriers in MoS_2_ channel. By utilizing the photo‐initiated carrier multiplication, their avalanche photodetectors exhibit the maximum responsivity of ≈3.4 × 10^7^ A W^−1^ and the detectivity of ≈4.3 × 10^16^ Jones under a low dark current, which are a few orders of magnitudes higher than the highest values reported previously, despite the absence of any additional chemical treatments or photosensitizing layers. The realization of both the ultrahigh photoresponsivity and detectivity is attributed to the interplay between the carrier multiplication by avalanche breakdown and carrier injection across a Schottky barrier between the channel and metal electrodes. This work presents a simple and powerful method to enhance the performance of photodetectors based on carrier multiplication phenomena in 2D materials and provides the underlying physics of atomically thin avalanche photodetectors.

## Graphical abstract

The authors demonstrate ultrasensitive MoS_2_ avalanche phototransistors by studying avalanche carrier multiplications under light illumination. By operating MoS_2_ transistors at a high source–drain voltage, they identify an optimal regime where photoresponsivity and detectivity simultaneously outperform previously reported values. Their work provides a powerful strategy to improve the performance of 2D‐materials‐based phototransistors and shows the potential of atomically thin avalanche photodetectors.

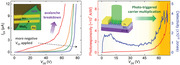

## 1 Introduction

Although the discovery of graphene pioneered the electronics and optoelectronics of 2D materials,^[^ ^1^ ^]^ its intrinsic lack of bandgap has limited the range of potential device applications.^[^ ^2^ ^]^ In contrast, 2D transition metal dichalcogenides (TMDCs) such as MoS_2_ and WSe_2_ possess both a bandgap and other alluring characteristics for semiconducting devices like high carrier mobility, good electrical stability, flexibility, and transparency.^[^ ^3^, ^4^ ^]^ Along with their excellent physical properties, numerous pragmatic methods to synthesize 2D TMDCs, including chemical vapor deposition (CVD) and gold‐mediated exfoliation, have been developed.^[^ ^5^, ^6^, ^7^ ^]^ Hence, they have been widely studied as a component of field‐effect transistors (FETs),^[^ ^8^, ^9^, ^10^ ^]^ logic circuits,^[^ ^11^ ^]^ and memory devices.^12^

In particular, their bandgap in the range of visible light and excellent photoconductivity triggered an extensive range of studies on 2D‐TMDC‐based optoelectronics and photonics such as light‐emitting diodes, solar cells, and single‐photon emitters.^[^ ^3^, ^13^, ^14^ ^]^ Following the first realization of MoS_2_ phototransistors,^[^ ^15^ ^]^ many groups have tried to improve the photoresponsive characteristics of 2D TMDC FETs. For instance, van der Waals (vdW) heterostructures comprised of 2D TMDCs and other photosensitizing layers like quantum dots and organometal halide perovskites showed dramatically enhanced photoresponsive characteristics.^[^ ^16^, ^17^ ^]^ Besides, the encapsulation of 2D TMDCs with high‐k materials such as HfO_2_ improved the photoresponsivity of MoS_2_ phototransistors up to ≈5 × 10^4^ A W^−1^.^[^ ^18^ ^]^ However, most of the previously reported phototransistors exhibited their highest demonstrated photoresponsivity in the on state of the FETs, thereby showing too high dark currents up to ≈100 µA that inevitably lead to large noise and low detectivity. Moreover, the incorporation of additional materials in the device architecture introduces extra complications in device fabrication and costs.

Meanwhile, avalanche breakdown phenomena have been widely utilized in a variety of ultrasensitive photodetectors like single‐photon avalanche diodes due to a high gain promised by carrier multiplication.^[^ ^19^ ^]^ The operation of photodetectors near the avalanche breakdown regime allows both a low dark current and ultrahigh photoresponsivity, which are essential for light‐sensing applications. Recently, the avalanche breakdown phenomena in MoS_2_ and other 2D materials have been observed,^[^ ^20^, ^21^, ^22^, ^23^ ^]^ which motivated us to anticipate demonstrating outstanding photoresponsive characteristics of MoS_2_ photodetectors based on carrier multiplication effects.

In this study, we fabricated ultrasensitive MoS_2_ avalanche phototransistors that could operate near the avalanche breakdown regime. All the MoS_2_ films used in this work were synthesized by CVD, which is relevant for future practical applications. By investigating the avalanche breakdown in the off state of the MoS_2_ FETs, we observed a different gate–source voltage dependence of the critical electric field at which the breakdown starts to occur compared to previous reports. Most interestingly, without capitalizing on any additional treatments or light absorption layers, the combination of avalanche carrier multiplication in the off state of the FETs and the excellent intrinsic photoconductivity of monolayer MoS_2_ let us dramatically improve the photoresponsivity and detectivity up to ≈3.4 × 10^7^ A W^−1^ and ≈4.3 × 10^16^ Jones, which are ≈60 times and ≈50 times larger values than the previous record, respectively, to the best of our knowledge. The maximum values of the photoresponsivity and external quantum efficiency (EQE) of the devices were ≈9.1 × 10^7^ A W^−1^ and ≈2.2 × 10^10^%, which potentially makes MoS_2_ avalanche phototransistors a promising candidate for atomically thin ultrasensitive photodetectors. We also showed that the performance of avalanche phototransistors could be tuned by controlling the contact barrier between the channel and contact metals.

## 2 Results and Discussion

**Figure 1:**
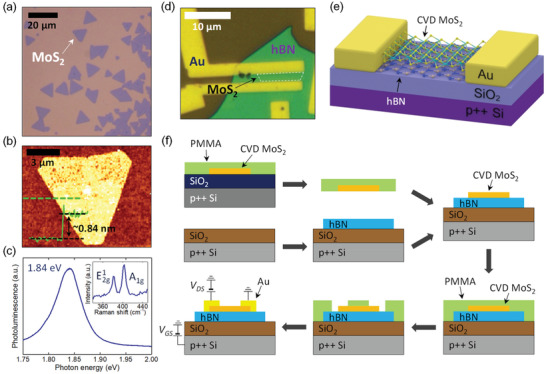
a) Optical image of CVD‐grown MoS_2_. b) AFM image of a CVD‐grown MoS_2_ flake with its topographic cross‐sectional profile across a green dashed line. c) PL and Raman (inset) spectra of MoS_2_. d) Optical image of a representative MoS_2_ FET fabricated on a hBN flake. e) Schematic of the fabricated MoS_2_ FET with Au contact. f) Fabrication procedure of MoS_2_ FETs.

All the MoS_2_ flakes used in this study were synthesized by CVD. The detailed process of the synthesis is provided in the Experimental Section. **Figure** **1**a shows the optical image of CVD‐grown MoS_2_ films. An atomic force microscopy (AFM) image of a MoS_2_ flake is demonstrated in Figure 1b with its topographic cross‐sectional profile. The thickness of the MoS_2_ film was estimated to be ≈0.84 nm, which corresponds to that of a monolayer.^[^ ^24^ ^]^ Figure 1c shows the photoluminescence (PL) and Raman spectra of a MoS_2_ film. Here, Raman peaks at ≈383 and ≈403 cm^−1^ correspond to the in‐plane (E^1^_2g_ mode) and the out‐of‐plane (A^1g^ mode) vibration modes of the monolayer MoS_2_, respectively.^[^ ^25^ ^]^ A sharp PL peak at the photon energy of ≈1.84 eV and the Raman shift difference of ≈20 cm^−1^ ensure a good quality of the CVD‐grown MoS_2_ films.^[^ ^24^, ^25^ ^]^ In this work, we fabricated MoS_2_ FETs on a mechanically exfoliated multilayer hBN flake. We note that the hBN flakes used in this study showed excellent crystalline properties, which is demonstrated in their Raman spectra (see Figure S1, Supporting Information). An optical image of the fabricated device is shown in Figure 1d, and its schematic illustration is given in Figure 1e. A heavily p‐doped Si with the resistivity of ≈5 × 10^−3^ Ω cm was used as a common back gate and Au metals served as source and drain electrodes. When fabricating the MoS_2_ FETs, we made use of the poly(methyl methacrylate) (PMMA)‐assisted transfer method to transfer MoS_2_ flakes onto hBN dielectric, for which the procedure is illustrated in Figure 1f. First, we spin‐coated a PMMA (11% concentration in anisole) film on a substrate where CVD MoS_2_ films were synthesized. We prepared another substrate on which hBN flakes were exfoliated. Then, we selected a specific region of MoS_2_ flakes to be used as the channel of a FET by using optical microscopy and transferred it onto the hBN flake. Finally, Au metal patterns (60 nm thick) were deposited on the MoS_2_/hBN heterostructure. Here, we chose the thickness of the metal patterns to be 60 nm so that contact breaking by the thickness of hBN flakes did not occur. A detailed device fabrication process is explained in the Experimental Section.

**Figure 2:**
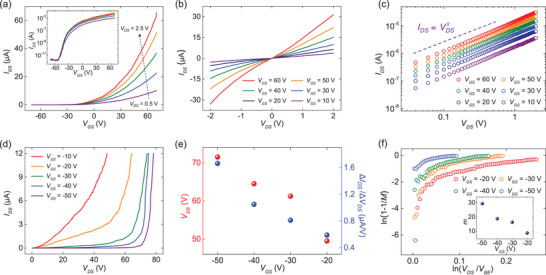
a) *I*_DS_–*V*_GS_ curves of the MoS_2_ FET at various drain–source voltages ranging from 0.5 to 2.5 V with a step of 0.5 V. The inset shows the same curves on the semilogarithmic scale. b) *I*_DS_–*V*_DS_ curves of the MoS_2_ FET. c) *I*_DS_–*V*_DS_ curves on the logarithmic scale of the MoS_2_ FET with an average *γ* value of 1.07. d) *I*_DS_–*V*_DS_ curves at various gate–source voltages ranging from −50 to −10 V with a step of 10 V. *V*_DS_ was swept from 0 to 78.25 V with a step of 0.25 V. e) *V*_EB_ and Δ*I*_DS_/Δ*V*_DS_ values versus *V*_GS_, represented as red and blue symbols, respectively. f) 1‐1/*M* values versus *V*_DS_ at various values of *V*_GS_. The inset shows the extracted values of *m* fitted using the Equation (2) at various values of *V*_GS_.

**Figure** **2**a,b demonstrates the transfer curve (source–drain current versus source–gate voltage; *I*_DS_–*V*_GS_ curve) and output curve (source–drain current versus source–drain voltage; *I*_DS_–*V*_DS_ curve) of the device shown in Figure 1d, respectively. Both of the curves were measured in vacuum (≈5 mTorr). The transfer curves showed a typical *n*‐type behavior with the on/off ratio of ≈3 × 10^7^. The threshold voltage of the FET was found to be ≈19 V. Here, we determined the threshold voltage to be the *x*‐axis intercept from the linear region of the transfer curves. In addition, the field‐effect mobility (*µ*) of the device was determined to be ≈54 cm^2^ V^−1^ s^−1^, as calculated by using the Equation (1):

(1) $$\mu \;=\left(\frac{\partial I_{\mathrm{DS}}}{\partial V_{\mathrm{GS}}}\right)\;\times \frac{L}{WC_{i}V_{\mathrm{DS}}}$$

with the channel length *L* = 1.9 µm, channel width *W* = 8.4 µm, and capacitance per unit area *C*_i_ = 10.5 nF cm^−2^. Herein, the dielectric constant of SiO_2_ (≈3.9) and bulk hBN (≈3.76)^[^ ^26^ ^]^ with the thickness values of the SiO_2_ layer (≈270 nm) and the hBN flake (≈56 nm) were used in the calculation of *C*_i_ (see Figure S1, Supporting Information, for the determination of the hBN flake thickness). The carrier mobility is higher than that of CVD‐grown MoS_2_ FETs directly fabricated on SiO_2_ without hBN, which is due to a weaker scattering from surface phonons and charged impurities owing to the absence of dangling bonds and charged surface states with the atomically flat characteristics of hBN.^[^ ^27^ ^]^ Moreover, we plotted the output curves in a logarithmic scale in Figure 2c to check the quality of contacts formed between the MoS_2_ channel and Au electrodes. The extracted average value of *γ* (linearity parameter in the output curve) was calculated to be 1.07, which suggests the formation of good contacts compared to devices exhibiting non‐linear output curves but not perfectly ohmic. It is known that the vdW gap between the channel and metal results in a nonzero contact barrier, even when the device shows good contact characteristics (see Figures S2 and S3, Supporting Information). As discussed later, this plays an important role in the performance of avalanche photodetectors.

To examine the electrical breakdown phenomena of MoS_2_ FETs, we measured the *I*_DS_ of the device shown in Figure 1d under high *V*_DS_ at various values of *V*_GS_ (see also Figure S4, Supporting Information). Figure 2d demonstrates the observed breakdown at diverse values of *V*_GS_. A drastic increase in *I*_DS_ along with *V*_DS_ can be clearly seen at *V*_GS_ = −20, −30, −40, and −50 V. Considering that other potential origins of the observed breakdown phenomena such as drain‐induced barrier lowering or thermal runaway can be neglected similarly according to our previous work,^[^ ^20^ ^]^ the observed breakdown phenomena can be attributed to avalanche multiplication, which is a carrier multiplication process that arises from collisions between high‐energy charge carriers and the lattice which create electron–hole pairs, thereby leading to an abrupt increase in *I*_DS_ (see Figure S5 and detailed discussions in Section S4, Supporting Information, for eliminating other potential artifacts). During these measurements, we set the compliance of *I*_DS_ as 12 µA so that the device does not become damaged by an excessive Joule heating. We confirmed that the device did not suffer from permanent damages by checking its electrical characteristics after each breakdown event, which showed no significant changes (see Figure S6, Supporting Information). We could also observe the same breakdown features in another MoS_2_ FET device with similar electrical characteristics (see Figure S7, Supporting Information). Here, we defined the electrical breakdown voltage (*V*_EB_) as *V*_DS_ value at which the breakdown starts to occur and the critical electric field (*E*_CR_) as *E*_CR_ = *V*_EB_/*L*. Moreover, Δ*I*_DS_/Δ*V*_DS_ can be defined as the average slope of *I*_DS_–*V*_DS_ curves after the onset of the breakdown. Interestingly, *V*_EB_ and Δ*I*_DS_/Δ*V*_DS_ were strongly dependent on the value of *V*_GS_ at which the breakdown was measured. The dependence of *V*_EB_ and Δ*I*_DS_/Δ*V*_DS_ on *V*_GS_ is shown in Figure 2e. The values of *V*_EB_ and Δ*I*_DS_/Δ*V*_DS_ decreased as *V*_GS_ increased from −50 to −20 V. On the other hand, a sharp increase in *I*_DS_ was not observable at *V*_GS_ = −10 V (see Figure 2d), which is attributable to a too large channel current (*I*_DS_) due to the gate‐field‐induced carriers before the avalanche breakdown can be observed. It can be seen that *I*_DS_ measured when *V*_GS_ = −10 V reached the compliance current at *V*_DS_ value of 48.5 V which is even below *V*_EB_ (49.5 V) for *V*_GS_ = −20 V. In overall, the observed dependence of *V*_EB_ on *V*_GS_ can be explained by the change of the contact barrier height between the channel and drain/source electrodes through the modulation of *V*_GS_. Please note that all the breakdown measurements were performed in the off state of the device (*V*_GS_ values between −50 and −20 V) since its threshold voltage was ≈19 V. In the off state of *n*‐type FETs, the height of the contact barrier increases as the applied *V*_GS_ becomes more negative.^[^ ^28^, ^29^ ^]^ Therefore, the higher value of *V*_DS_ is required for charge injection and to observe the onset of the electrical breakdown. Since electrons injected from the source to MoS_2_ feel larger electric fields in the channel as *V*_DS_ increases, the value of Δ*I*_DS_/Δ*V*_DS_ increases with an increase in *V*_EB_, that is, a decrease in *V*_GS_. The details of the mechanism behind these observations will be discussed later.

From the obtained electrical characteristics, we could also analyze the underlying characteristics of breakdown phenomena in MoS_2_ FETs. The multiplication factor, which means how large a channel current was produced by the electrical breakdown, is defined as *M*(*V*_DS_) = *I*_DS_(*V*_DS_)/*I*_DS_(*V*_DS_ = *V*_EB_). We note that the multiplication factor is a function of *V*_DS_. Then, we plotted 1‐1/*M* as a function of *V*_DS_/*V*_EB_ at different *V*_GS_ values, as shown in Figure 2f. Empirically, the relationship between 1‐1/*M* and *V*_DS_/*V*_EB_ is given by the Equation (2):

(2) $$1-\;\frac{1}{M}={\left(\frac{V_{\mathrm{DS}}}{V_{\mathrm{EB}}}\right)}^{m}\;$$

where *m* can be obtained by fitting.^[^ ^21^ ^]^ Here, the fitting was performed near ln(*V*_DS_/*V*_EB_) value of 0.05, namely just after the onset of the breakdown, similarly to previous works.^[^ ^21^, ^23^ ^]^ The obtained values of *m* are shown in the inset of Figure 2f. As a result, *m* increased as *V*_GS_ decreased from −20 to −50 V. This is in an agreement with the result shown in Figure 2e in that the value of Δ*I*_DS_/Δ*V*_DS_ gets larger as *m* increases. From the multiplication factor, we could calculate the impact ionization rate of electrons (*α*) by using the Equation (3):

(3) $$\alpha \left(E\right)\;=\frac{1}{n}\;\;\frac{dn}{dx}=\frac{1}{L}\;\left(1-\frac{1}{M}\right)$$

where *n* is the electron density and *L* is the channel length.^[^ ^30^, ^31^ ^]^ The impact ionization rate *α*(*E*) indicates the ionization per unit path length at an electric field of *E* (see Figure S6 and related discussions in Section S5, Supporting Information, for the details of the Equation (3)). Note that *α* is a function of the applied electric field *E*, which is determined by *V*_DS_. The calculated values of *α* are demonstrated in Figure S6, Supporting Information.

**Figure 3:**
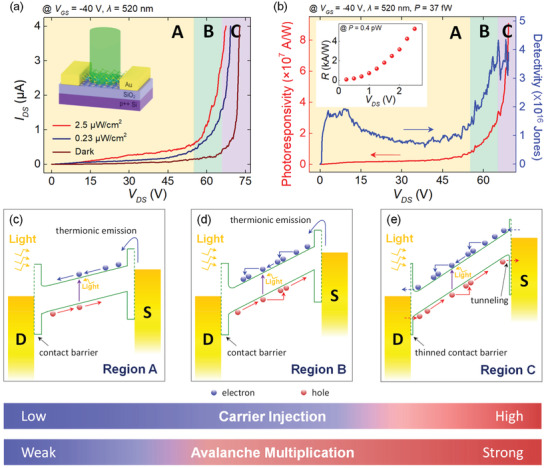
a) *I*_DS_ measured in the dark and under the laser irradiation with different irradiation intensities. *V*_DS_ was swept from 0 to 72.75 V with a step of 0.25 V. The inset shows a schematic illustration of laser irradiation to a MoS_2_ FET. b) Photoresponsivity and detectivity calculated under the irradiation with the intensity of 0.23 µW cm^−2^ versus *V*_DS_. The inset shows the photoresponsivity (*R*) of the same device calculated under the irradiation with the intensity of 2.5 µW cm^−2^ at *V*_DS_ from 0.25 to 2.5 V. Schematics of energy band diagrams in the off state of MoS_2_ FETs in the c) region A, d) region B, and e) region C. Red and blue symbols indicate electrons and holes, respectively. S and D denote source and drain, respectively. Two colored bands (bottom) indicate the *V*_DS_ region where carrier injection and avalanche multiplication are weak (blue) or strong (red), respectively.

Next, we characterized the photoresponse of MoS_2_ FETs over the avalanche breakdown regime at high drain–source biases by illuminating the FET channel with a laser. **Figure** **3**a shows the channel current measured in the dark (*I*_dark_) and under the laser irradiation (*I*_irra_) at wavelength of 520 nm and different laser intensities (0.23 and 2.5 µW cm^−2^). In this device, we chose the value of *V*_GS_ to be −40 V since the dark current at this gate–source voltage was low enough to be suitable for a highly sensitive photodetection (see Figure 2a). *I*_irra_ showed the electrical breakdown at a voltage lower than the onset voltage of the dark current's breakdown, and the output curves obtained under illumination were less steep, suggesting that *m* is smaller under illumination than in the dark. Similarly to the dependence of Δ*I*_DS_/Δ*V*_DS_ on *V*_GS_ for the breakdown of dark currents (see Figure 2e), this can be attributed to a smaller value of an electric field that charge carriers feel in the channel arising from the earlier onset of breakdown behavior (i.e., at a smaller *V*_DS_). This is consistent with the obtained values of *m* (see Figure S8, Supporting Information). As a result, *V*_DS_ can be classified into three regions, according to Figure 3a. Below the *V*_DS_ value of ≈54 V (region A, yellow boxes in Figure 3a,b), no evident breakdown phenomena were observed in both the currents measured in the dark and under the laser irradiation. When 54 V <*V*_DS_ < 66 V (region B, green boxes in Figure 3a,b), only the *I*_irra_ abruptly increased as *V*_DS_ increased whereas the dark current did not exhibit such a drastic increase. Above the *V*_DS_ value of ≈66 V (region C, purple boxes in Figure 3a,b), both of the currents showed a rapid increase with an increase in *V*_DS_. We confirmed that this phenomenon is not attributed to the extrinsic origins in the devices such as charge accumulation at the interface or trap sites by measuring the photoresponse with various illumination times (see Figure S9, Supporting Information). On the other hand, the measured photocurrent versus laser irradiation power became highly nonlinear as *V*_DS_ increased, which can be attributed to the avalanche breakdown effect (see Section S9, Supporting Information). Moreover, the same behavior was observed in another device when illuminated with different wavelengths (405, 520, and 658 nm), which demonstrates the generality of the observed behavior within a broad range of visible light spectrum (see Figure S11, Supporting Information). We note that the operation of MoS_2_ avalanche phototransistors in the region B is desirable for achieving both a stable operation and sensitive photodetection due to the low dark current (≈100 nA), the absence of the electrical breakdown in the dark current (i.e., device operation stability in dark), and a large photocurrent up to ≈1.5 µA under a low laser intensity. Meanwhile, the operation in the region C would be at a relative disadvantage because the dark current increases rapidly with *V*_DS_, which obstructs the stable operation of the device. In case of the MoS_2_ FET with Au contact, the voltage window of the region B was ≈12 V, which corresponds to ≈6.3 × 10^−2^ MV cm^−1^ of electric field. The division of *V*_DS_ into the three regions has similarly been reported in avalanche phototransistors based on other materials.^[^ ^32^, ^33^ ^]^ The reason *V*_DS_ can be divided into these three regions originates from a contact barrier between MoS_2_ and Au electrodes, which will be discussed later in more details.

The device performance of photodetectors is typically evaluated by their photoresponsivity, detectivity, and EQE. Photoresponsivity represents how large a photocurrent is produced by the incident irradiation. In contrast with photoresponsivity, detectivity takes account of the magnitude of photoresponsivity with respect to both the incident irradiation and the noise level of the dark current. EQE represents how many charge carriers are created by one incident photon. In general, photoresponsivity (*R*), detectivity (D^*^), and EQE are defined as the Equations (4)–(6):

(4) $$R\;=\frac{I_{\mathrm{ph}}}{P}\;$$

(5) $$D^{*}=\frac{{\left(A_{D}\Delta f\right)}^{1/2}}{\mathrm{NEP}}\;$$

(6) $$\mathrm{EQE}\;=\frac{I_{\mathrm{ph}}/q}{P/\left(h\nu \right)}\;=\;R\left(\frac{h\nu }{q}\right)$$

where *I*_ph_ is the photocurrent, *P* is the intensity of the incident light, *A*_D_ is the effective detection area of the device, Δ*f* is the electrical bandwidth, NEP is noise equivalent power, *q* is the elementary charge ( = 1.602 × 10^−19^ C), h is the Planck constant (=6.626 × 10^−34^ J s), and *ν* is the frequency of the incident light. Assuming that the shot noise is a dominant source of the current noise, the detectivity in the Equation (5) can be expressed as $D^{*}=RA_{D}^{1/2}/\left\langle i_{\mathrm{shot}}^{2}\right\rangle \;$, where $\left\langle i_{\mathrm{shot}}^{2}\right\rangle =\sqrt{2qI_{\mathrm{DS}}}\;$ in the region A and B and $\left\langle i_{\mathrm{shot}}^{2}\right\rangle =\sqrt{2qI_{\mathrm{DS}}M^{2}F\left(M\right)}\;$ in the region C. Here, *M* is the multiplication factor and $F\;\left(M\right)=\;kM+\left(1-k\right)\left(2-\frac{1}{M}\right)$ where *k* ∈ (0,  1),^[^ ^34^ ^]^ which results from the intrinsic random nature of avalanche multiplication processes. In the following results, detectivity in the region C was calculated at *k*  =  0.5 because it has been shown that the choice of *k* does not significantly affect the values of detectivity.^[^ ^21^ ^]^ The calculated values of photoresponsivity and detectivity under the laser intensity of 0.23 µW cm^−2^ as functions of *V*_DS_ are plotted in Figure 3b. The corresponding values calculated at the laser intensity of 2.5 µW cm^−2^ are shown in Figure S8, Supporting Information. Both the photoresponsivity and detectivity nearly saturated when 30 V < *V*_DS_ < 54 V but drastically increased in the regions B. We stress that the maximum values of photoresponsivity, detectivity, and EQE in the region B were obtained as ≈3.4 × 10^7^ A W^−1^, ≈4.3 × 10^16^ Jones and ≈8.1 × 10^9^%, respectively. To the best of our knowledge, this value of photoresponsivity is ≈60 times higher than the previous record reported for MoS_2_ photodetectors, and the detectivity is ≈50 times higher than the previously reported values.^[^ ^35^ ^]^ It should be noted that this FET exhibited the photoresponsivity comparable to previously reported devices in a low‐*V*_DS_ regime (≈1.81 × 10^3^ A W^−1^ at *V*_DS_ = 1.5 V and *P* = 0.4 pW).^[^ ^36^, ^37^, ^38^ ^]^ For example, a similar MoS_2_ FET with Au contacts was demonstrated to have the photoresponsivity of ≈5 × 10^3^ A W^−1^ at *V*_DS_ = 5 V and *P* = 4 pW in the off state.^[^ ^38^ ^]^ This implies that the ultrasensitive characteristics of the device primarily originate from the avalanche breakdown. Moreover, these values were obtained under a relatively low dark current (≈100 nA) enabled by the existence of a contact barrier, whereas almost all of the sensitive MoS_2_ phototransistors reported previously exhibited their high photoresponsivity in the on state under a high dark current.^[^ ^16^, ^18^ ^]^ This definitely shows an enormous potential and extensive availability of MoS_2_ avalanche phototransistors in ultrasensitive 2D optoelectronics. Here, we note that monolayer MoS_2_ is a promising candidate for the realization of ultrasensitive 2D avalanche photodetectors, in that previously reported avalanche phototransistors based on other 2D materials like black phosphorous and InSe exhibited several orders of magnitude lower photoresponsivity and detectivity.^[^ ^23^, ^33^ ^]^ We believe that further improvements on the electrical properties of MoS_2_ devices should enable the realization of more sensitive MoS_2_ avalanche phototransistors, in that higher values of field‐effect mobility in general enhance optoelectronic figures of merit and 2D TMDC FETs have demonstrated better transport characteristics in several previous reports (see Section S11, Supporting Information). In addition, the maximum values of photoresponsivity, detectivity, and EQE in the region C were found to be ≈9.1 × 10^7^ A W^−1^, ≈4.3 × 10^16^ Jones, and ≈2.2 × 10^10^%, respectively. It should be noted that there was no clear increase in detectivity in the region C due to the noise contribution from the random nature of the avalanche multiplication process, which is taken into account in the calculation of detectivity. Although these values of photoresponsivity and EQE could be practically less relevant than those of the region B unless a stable operation within the region C can be guaranteed, the photoresponsivity and detectivity values are ≈150 and ≈50 times larger than the former record, respectively.^[^ ^35^ ^]^

The obtained electrical and optoelectronic characteristics of the MoS_2_ avalanche phototransistors can be explained by considering energy band alignment. The energy band diagrams corresponding to the region A, B, and C are illustrated in Figures 3c, 3d, and 3e, respectively. As discussed earlier, not only the difference between the work function of MoS_2_ and Au but also the vdW gap between MoS_2_ and Au contributes to a contact barrier in MoS_2_ FETs, as represented in the band diagrams.^[^ ^39^ ^]^ Due to this barrier, the output curves at low temperatures usually exhibit the characteristics of a non‐ohmic contact, even if they appear ohmic at room temperature due to a sufficient thermal energy available (see Figures S2 and S3, Supporting Information).^[^ ^40^ ^]^ In the region A (Figure 3c), the electric field in the MoS_2_ channel produced by the drain–source voltage is not strong enough to induce carrier multiplication. Thus, the breakdown does not occur either in the dark or under illumination. In case of the region B (Figure 3d), photogenerated carriers can undergo impact ionization provided that the applied field is strong enough to initiate the avalanche breakdown. Hence, this process results in a drastic increase in *I*_irra_. However, the contact barrier is still too thick for electrons and holes to be injected into the MoS_2_ channel. Therefore, the breakdown does not occur under the dark condition. In addition, *V*_EB_ increases as *V*_GS_ decreases (see Figure 2e) since the contact barrier height that hinders carrier injection increases with a decrease in *V*_GS_. In the region C (Figure 3e), the external electric field is strong enough to induce the avalanche breakdown and the contact barrier also becomes sufficiently thin due to drain‐induced barrier thinning, and thus electrons and holes can be easily injected into the channel by quantum‐mechanical tunneling.^[^ ^33^, ^41^ ^]^ Therefore, the breakdown can occur both in the dark and light conditions in the region C. Our interpretation based on the energy band diagrams outlined above can be further supported by examining avalanche breakdown in Pd‐contact MoS_2_ FETs, expected to have higher contact barrier than Au‐contact devices,^[^ ^42^, ^43^ ^]^ in dark and under light illumination, respectively (see Section S12, Supporting Information, for detailed discussions). First, a larger critical electric field for dark currents (i.e., *E*_CR,dark_) of the Pd‐contact device (≈0.46 MV cm^−1^, see Figure S12, Supporting Information) than that of the Au‐contact device (between ≈0.26 and ≈0.37 MV cm^−1^ in the *V*_GS_ range from −20 to −50 V) can be attributed to the higher contact barrier in the Pd‐contact devices, which delays the *V*_DS_ onset of the electrical breakdown in the dark condition. Second, a comparable critical field for *I*_irra_ (i.e., *E*_CR,irra_) of ≈0.34 MV cm^−1^ under light illumination (see Figure S13, Supporting Information) to ≈0.29 MV cm^−1^ for the Au‐contact device (Figure 3a), demonstrates that the breakdown under light illumination is hardly affected by the contact barrier. This supports that the breakdown in the region B was triggered by the photogenerated carriers rather than the electrically injected carriers. In overall, the comparative study with the Pd‐contact MoS_2_ FETs confirms the role of the contact barrier in determining the voltage range for optimal operation of the avalanche phototransistors, in addition to providing a potential tunability of the stable voltage operation range.

**Figure 4:**
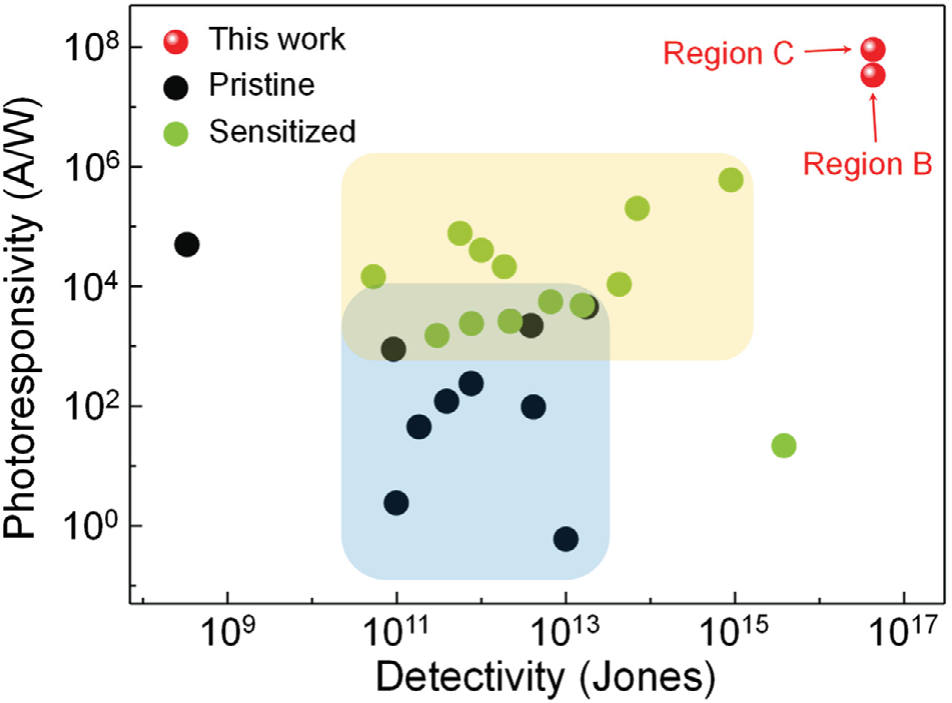
Photoresponsivity and detectivity of our phototransistor devices and previously reported devices based on 2D TMDCs. Note that the device performance parameters in this work (i.e., photoresponsivity and detectivity) were characterized by using the same method as in the literature. A red symbol written with region B and region C indicates the maximum values of photoresponsivity and detectivity of our device in the region B and region C, respectively. The data of pristine phototransistors were obtained from references: MoS_2_,^[^ ^18^, ^36^, ^37^ ^]^ MoSe_2_,^[^ ^44^, ^45^ ^]^ WS_2_,^[^ ^46^, ^47^, ^48^, ^49^ ^]^ and WSe_2_.^[^ ^50^ ^]^ The data of sensitized phototransistors were obtained from references: devices treated with self‐assembled monolayers,^[^ ^17^, ^51^ ^]^ photosensitized with quantum dots,^[^ ^16^, ^35^, ^52^, ^53^, ^54^ ^]^ organometal halide perovskites,^[^ ^17^, ^55^, ^56^ ^]^ oxides,^[^ ^57^, ^58^ ^]^ and ferroelectrics.^[^ ^59^ ^]^ The blue and yellow areas indicate the region where most pristine (black symbols) and sensitized photodetectors (green symbols) that have been reported in literature occupy, respectively.

The comparison of the photoresponsivity and detectivity values of our devices with the performance of other photodetectors is provided in **Figure** **4**.^[^ ^16^, ^17^, ^18^, ^35^, ^36^, ^37^, ^44^, ^45^, ^46^, ^47^, ^48^, ^49^, ^50^, ^51^, ^52^, ^53^, ^54^, ^55^, ^56^, ^57^, ^58^, ^59^ ^]^ We compared our devices to state‐of‐the‐art phototransistors based on 2D TMDCs, for which the device performance was characterized in a consistent way with our avalanche phototransistors. In addition, the comparison of photoswitching dynamics is demonstrated in Table S2, Supporting Information (see Section S14, Supporting Information, for further notes), where the photoswitching behavior measured in this work showed the rise and decay time in the range of 4.1 to 27 s and 0.2 to 2.9 s, respectively. Although these values are comparable or smaller than various previous reports on 2D phototransistors (see Section S14, Supporting Information), ultrafast photoswitching dynamics in a microsecond time scale have also been demonstrated in 2D‐TMDC‐based phototransistors.^[^ ^47^, ^58^ ^]^ We believe that further optimizations on the quality of MoS_2_ films and MoS_2_/dielectric interfaces can decrease the number of trap sites, which will make it possible to enhance the photoswitching characteristics in that the photoswitching dynamics of 2D phototransistors are often influenced by the photogating effect (see Section S15, Supporting Information).^[^ ^18^, ^60^, ^61^ ^]^ In Figure 4, the phototransistors are classified into two types, namely pristine and sensitized phototransistors. In case of pristine phototransistors, their semiconductor channels purely consist of a single type of 2D TMDC materials, whereas the sensitized phototransistors are those chemically treated with organic molecules such as (3‐aminopropyl)triethoxysaline or octadecyltrichlorosilane, or comprised of a hybrid structure with light‐sensitive materials like quantum dots or organometal halide perovskites. Here, we did not attempt to compare our phototransistor devices to photodiodes because photodiodes have different device architectures. In general, phototransistors based on 2D TMDCs show higher photoresponsivity and EQE than photodiodes due to their photogating effect (see Figure S17, Supporting Information).^[^ ^18^, ^60^, ^61^ ^]^ It is evident from Figure 4 that our devices exhibited much higher performance than other devices in terms of both photoresponsivity and detectivity, even though we did not incorporate any chemical treatments or additional photosensitizing layers. We stress that it was possible to improve both photoresponsivity and detectivity at the same time by employing the avalanche multiplication of photogenerated carriers while suppressing the dark current in the off state of the FET. Moreover, we demonstrated the possibility of a stable and sensitive photodetection of the avalanche photodetectors by controlling the carrier injection at the 2D‐metal contact. We believe that phototransistors based on TMDCs such as MoSe_2_, which show lower photoresponsivity in comparison to photodetectors based on MoS_2_, WS_2_, or WSe_2_^[^ ^44^, ^45^ ^]^ can also be greatly improved by taking advantage of the avalanche breakdown since a vdW gap generated at TMDC‐metal contact is a general property of 2D electronic devices.^[^ ^39^ ^]^

## 3 Conclusion

In conclusion, we fabricated MoS_2_ avalanche phototransistors on hBN flakes and investigated their electrical and optoelectronic characteristics. The onset of the avalanche breakdown at high drain–source voltages could be modulated by the gate‐voltage‐dependent carrier injection through the change in a contact barrier between the MoS_2_ channel and drain/source electrodes. When the devices were characterized under illumination, the breakdown began at a lower drain–source bias, which supports the initiation of the breakdown by photogenerated carriers in the channel. The carrier multiplication of these photogenerated carriers manifests itself to excellent photoresponsivity, detectivity and EQE of our avalanche phototransistors which were ≈3.4 × 10^7^ A W^−1^, ≈4.3 × 10^16^ Jones, and ≈8.1 × 10^9^%, respectively, under a low dark current. These values were a few orders of magnitude higher than the previously reported values of 2D‐TMDC‐based photodetectors even though we did not employ any additional chemical treatments or photosensitizing layers. The obtained values of EQE make MoS_2_ avalanche phototransistors a promising candidate for 2D single‐photon detectors. Overall, we demonstrated that the interplay between carrier multiplication by avalanche breakdown and carrier injection by the contact barrier plays a pivotal role in the simultaneous enhancement of the photoresponsivity and detectivity of MoS_2_ avalanche phototransistors. Our work proposes a simple and powerful strategy to improve the performance of 2D‐TMDC‐based phototransistors and grants a deeper understanding of atomically thin avalanche photodetectors, which are a relatively unexplored area of research in 2D optoelectronics.

## 4 Experimental Section

4.1

4.1.1

##### MoS_2_ Thin Film Synthesis

The uniform monolayer MoS_2_ films were grown on a SiO_2_/Si substrate which had 270‐nm‐thick SiO_2_ thermally grown on the heavily p‐doped Si layer. The authors used a dual‐heating zone CVD system (Teraleader Co., Korea) for the growth of MoS_2_. Here, the substrate and boat containing MoO_3_ powder were heated up to ≈750 °C and the other boat with sulfur powder was heated up to ≈250 °C. Ar gas was used as a carrier gas.

##### Fabrication of MoS_2_ FETs

First, the authors prepared two substrates with a 270‐nm‐thick SiO_2_ layer on a heavily doped p++ Si wafer. Then, hBN flakes were mechanically exfoliated from a bulk hBN crystal (SPI supplies) on one of the substrates. The other substrate was used in the CVD synthesis of monolayer MoS_2_ films. To make MoS_2_/hBN heterostructures, they transferred MoS_2_ onto a hBN flake by using the poly(methyl methacrylate) (PMMA)‐assisted transfer method as shown in Figure 1f. Subsequently, they spin‐coated PMMA (11% concentration in anisole) as an electron resist layer at 4000 rpm to create drain/source electrode patterns. After the spin coating, the samples were baked on a hot plate at 180 °C for 150 s. They designed the electrode patterns using an electron‐beam lithography system (JSM‐6610, JEOL) and developed the patterns with a methyl isobutyl ketone/isopropyl alcohol (1:3) solution for 50 s. Finally, the Au or Pd metal (60 nm thick) patterns were deposited with an electron‐beam evaporator system (KVE‐2004L, Korea Vacuum Tech). The deposited metal was lifted off by acetone with the lift‐off time of 600 s.

##### Material and Device Characterization

The thickness of materials was measured by using an atomic force microscopy (NX 10 AFM, Park Systems). The PL and Raman spectra were obtained through a confocal imaging system (XperRaman 200, Nanobase) with the incident laser beam of wavelength 532 nm. The electrical characteristics of FETs were measured by using a probe station (M6VC, MS TECH) and a semiconductor parameter analyzer (Keithley 4200, USA). All of the electrical breakdown measurements were performed in the ambient environment. The photoresponses of FETs were measured under the laser (MDE5240V, Korea) illumination of wavelength 520 nm, if the wavelength was not specifically denoted. The laser beam was globally illuminated to devices with the diameter of few millimeters. All the characterizations were performed at room temperature unless the measurement temperature was specifically indicated.

## Conflict of Interest

The authors declare no conflict of interest.

## Supporting information

Supporting Information

## Data Availability

Research data are not shared.
