# Supplementary material for: Ultrasensitive Photodetection in MoS2 Avalanche Phototransistors
Source: Adv Sci (Weinh). 2021 Aug 8;8(19):2102437. doi: 10.1002/advs.202102437 (PMC8498866; doi:10.1002/advs.202102437)
Supplement: Supplementary file 1 — Supporting Information [file ADVS-8-2102437-s001.pdf]

© 2021 Wiley-VCH GmbH

ADVANCED SCIENCE
Open Access

Supporting Information

for *Adv. Sci.*, DOI: 10.1002/advs.202102437

Ultrasensitive Photodetection in MoS2 Avalanche Phototransistors

Junseok Seo, Jin Hee Lee, Jinsu Pak, Kyungjune Cho, Jae-Keun Kim, Jaeyoung Kim, Juntae Jang, Heebeom Ahn, Seong Chu Lim, Seungjun Chung, Keehoon Kang and Takhee Lee**

Supporting Information

Ultrasensitive Photodetection in MoS_2 Avalanche Phototransistors

Junseok Seo, Jin Hee Lee, Jinsu Pak, Kyungjune Cho, Jae-Keun Kim, Jaeyoung Kim, Juntae Jang, Heebeom Ahn, Seong Chu Lim, Seungjun Chung, Keehoon Kang and Takhee Lee**

J. Seo, Dr. J. Pak, J. Kim, J. Jang, H. Ahn, Prof. T. Lee
Department of Physics and Astronomy, and Institute of Applied Physics, Seoul National University, Seoul 08826, Korea

J. H. Lee
Department of Energy Science, Sungkyunkwan University, Suwon 16149, Korea
Center for Integrated Nanostructure Physics, Institute for Basic Science (IBS), Sungkyunkwan University, Suwon 16149, Korea

Dr. K. Cho
Soft Hybrid Materials Research Center, Korea Institute of Science and Technology, Seoul 02792, Korea

Dr. J.-K. Kim
Max-Planck Institute of Microstructure Physics, 06120 Halle, Saale, Germany

Prof. S. C. Lim
Department of Energy Science, Sungkyunkwan University, Suwon 16149, Korea
Department of Smart Fabrication Technology, Sungkyunkwan University, Suwon 16149, Korea

Dr. S. Chung
Soft Hybrid Materials Research Center, Korea Institute of Science and Technology, Seoul 02792, Korea

KHU-KIST Department of Converging Science and Technology, Kyung Hee University, Seoul 02447, Korea

Prof. K. Kang
Department of Materials Science and Engineering, Yonsei University, Seoul 03722, Korea

*E-mail: tlee@snu.ac.kr; keehoon.kang@yonsei.ac.kr

Keywords: Transition metal dichalcogenide, field-effect transistor, electrical breakdown, avalanche photodetector, photoresponsivity

Table of Contents

1. Hexagonal boron nitride flake
2. Electrical characteristics of MoS_2 FETs at low temperatures
3. Electrical breakdown measured in different environments
4. Mechanism of an abrupt increase in I_{DS}
5. Absence of device degradation and calculation of impact ionization rates
6. Electrical characteristics and breakdown of another MoS_2 FET
7. V_{DS}-dependent photoresponsivity and detectivity under high illumination intensity
8. Illumination-time-dependent measurement of photo-initiated carrier multiplication
9. Linearity of photocurrents
10. Photo-initiated carrier multiplication under illumination with different wavelengths
11. Comparison of field-effect mobility
12. MoS_2 avalanche phototransistors with Pd contact
    A. Electrical characteristics of the MoS_2 FET with Pd contact
    B. Electrical breakdown of the MoS_2 FET with Pd contact
    C. Photoresponse of the MoS_2 FET with Pd contact under various laser intensities
    D. Physical explanation of Pd-contact MoS_2 avalanche phototransistors
13. Contact resistance of the MoS_2 FET with Au and Pd contact
14. Characterization and comparison of photoswitching response times
15. Photogating effect of MoS_2 FETs

References

1. Hexagonal boron nitride flake

Figure S1a shows the Raman spectra of the hBN flake used in this study. The Raman peak at ~1360 cm^{-1} demonstrates the good crystalline quality of the employed flake.[S1] The inset of Figure S1a shows the corresponding vibration mode of the measured Raman peak. Figure S1b shows the AFM image of the hBN flake used in the device demonstrated in Figure 1d in the main manuscript. The topographic cross-sectional profile (inset of Figure S1b) shows that the thickness of the flake is ~56 nm.

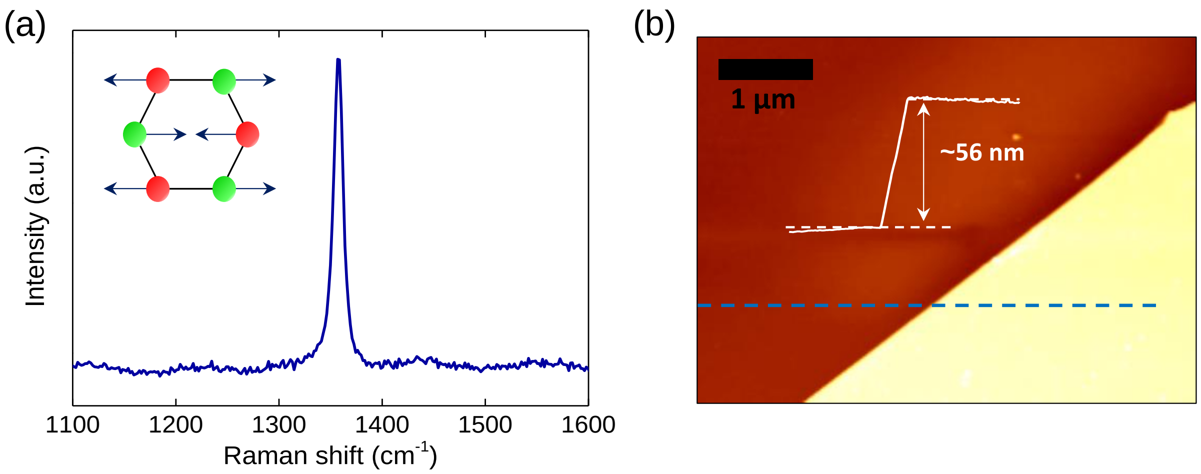

Figure S1. (a) Raman spectra of the hBN flake shown in Figure 1d in the main manuscript. The inset figure shows the vibration mode of the corresponding Raman peak. The red and green symbols indicate a boron and nitrogen atom, respectively. (b) AFM image of the hBN flake shown in Figure 1d with its topographic cross-sectional profile along the dashed line.

2. Electrical characteristics of MoS_2 FETs at low temperatures

Although a vdW gap between the channel and contact metals results in a finite contact barrier, output curves measured at room temperature can show a linear shape. To show this point, we studied the low-temperature electrical characteristics of MoS_2 FETs. We fabricated a MoS_2 FET on a hexagonal boron nitride. The device is shown in the inset of Figure S2a, whose transfer curve measured at the temperature of 300 K is shown in Figure S2a. The transfer curve shows that the device exhibited a typical n-type behavior. Then, we measured the electrical characteristics of the device. According to Figure S2b, I_{DS} increased as the temperature increased from 80 K to 260 K, which is typical of the thermionic emission. From I_{DS} measured in each V_{GS} and temperature, we could estimate the V_{GS}-dependent contact barrier φ from the equation (S1) in the standard thermionic emission theory,[S2]

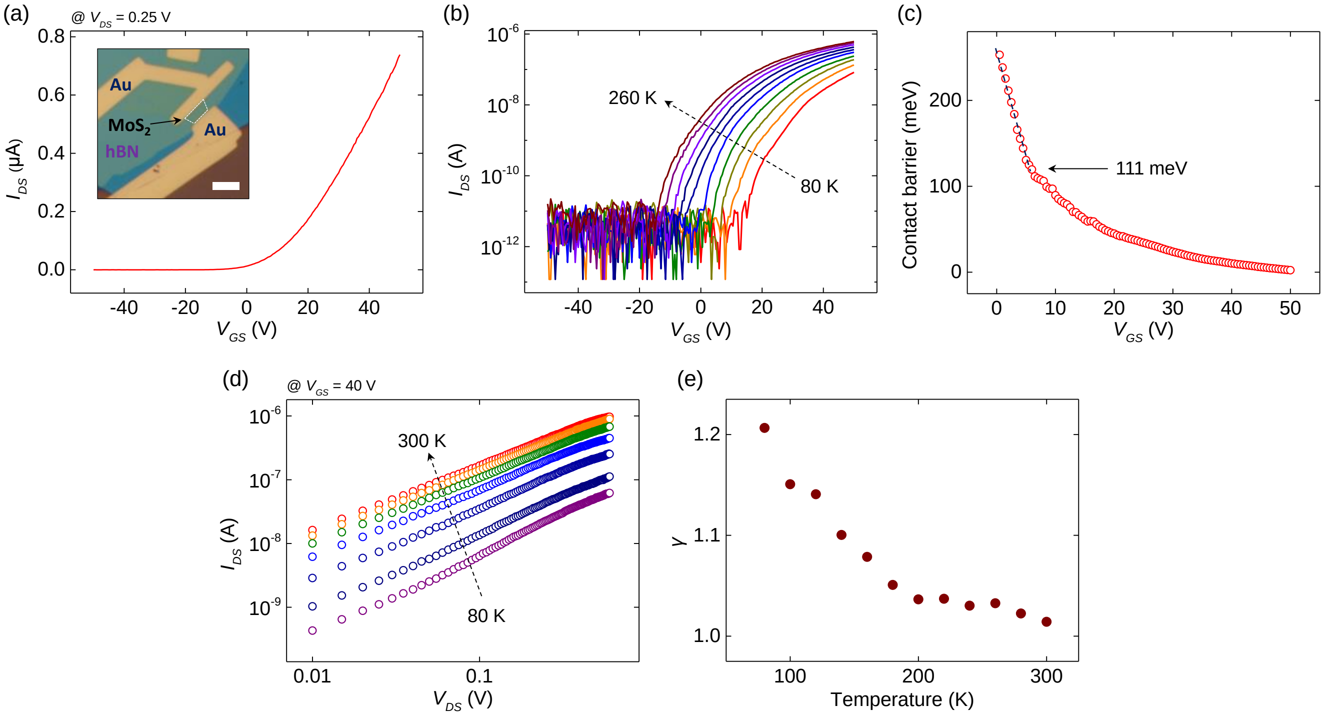

Figure S2. (a) Output curve at room temperature of the device shown in the inset. Scale bar: 3 μm. (b) Transfer curves measured at temperatures from 80 to 260 K with the step of 20 K. All the curves were measured at V_{DS}=0.25 V. (c) V_{GS}-dependent contact barrier estimated by using the equation (S1). (d) Output curves measured at temperatures of 80, 100, 140, 180, 220, 260 and 300 K. (e) Linearity parameter γ extracted from the output curves.

$$I_{DS} = SA^*T^{3/2}e^{-q\varphi/k_BT}\left(1 - e^{-qV_{DS}/nk_BT}\right) \qquad \text{(S1)}$$

where S is the contact area, $A^* = \frac{4qk_B^2m^*}{h^3}$ is the Richardson coefficient, m^* is an effective mass, T is a temperature, and n is an ideality factor. The calculated V_{GS}-dependent contact barrier is demonstrated in Figure S2c. Please note that the contact barrier decreases as V_{GS} increases, which was observed in several reports.[S3,S4] Furthermore, the Schottky barrier of this device was determined to be ~111 meV from Figure S2c, which is in agreement with that of previous works that studied MoS_2/Au contact.[S4,S5] Even though this indicates a nonzero value of a Schottky barrier, the thermal energy of electrons at room temperature is sufficient to overcome this energy barrier. In this regard, the existence of a finite Schottky barrier can be in agreement with linear-shaped output curves. In addition, this implies that output curves become more S-shaped (i.e. linearity parameter γ in the relation $I_{DS} \propto V_{DS}^{\gamma}$ deviates more from 1) as temperature goes down due to the lack of electrons' thermal energy, which are shown in Figures S2d and S2e.

In addition, we tested another device to investigate the effect of temperatures. Figures S3a and S3b illustrate the output curves of the device shown in the inset of Figure S3a at the temperature of 300 and 80 K, respectively. Although the device exhibited an ohmic-contact property at T = 300 K, it also showed a weak non-ohmic contact at T = 80 K, which is in agreement with the results in Figure S2. Figures S3c and S3d show the breakdown at T = 300 K and 80 K, respectively. The breakdown started at the lower value of V_{DS} at a lower temperature, which is consistent with the previous reports.[S6]

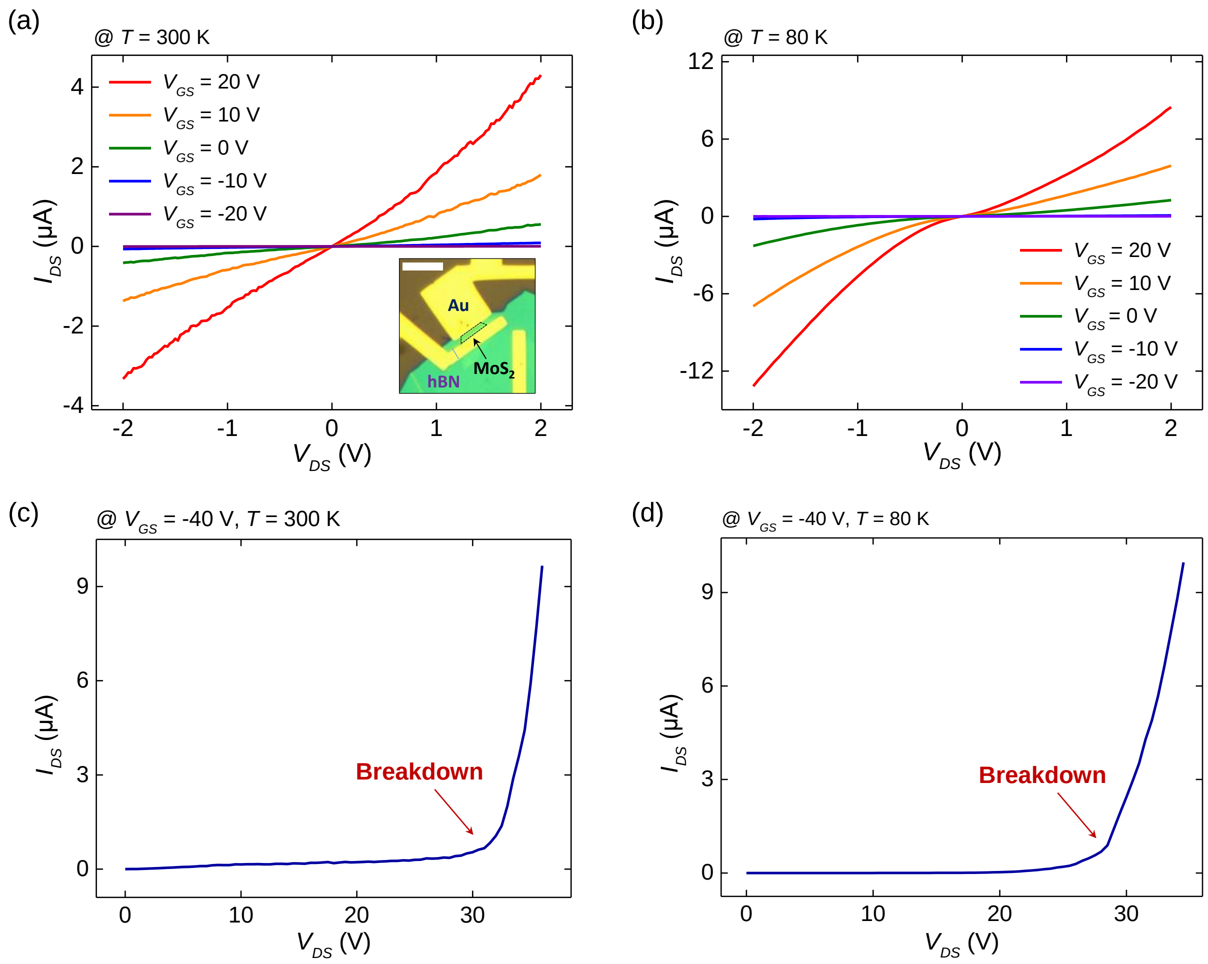

Figure S3. The output curve of the device shown in the inset of (a) at the temperature of (a) 300 K and (b) 80 K. The electrical breakdown measured at V_{GS} = -40 V and the temperature of (c) 300 K and (b) 80 K.

3. Electrical breakdown measured in different environments

Figure S4 shows the avalanche breakdown measured in the ambient and N_2 environment. We note that the breakdown measured in the ambient environment showed considerably different characteristics to that measured in N_2 environment, which signifies the role of the environment in the breakdown process.

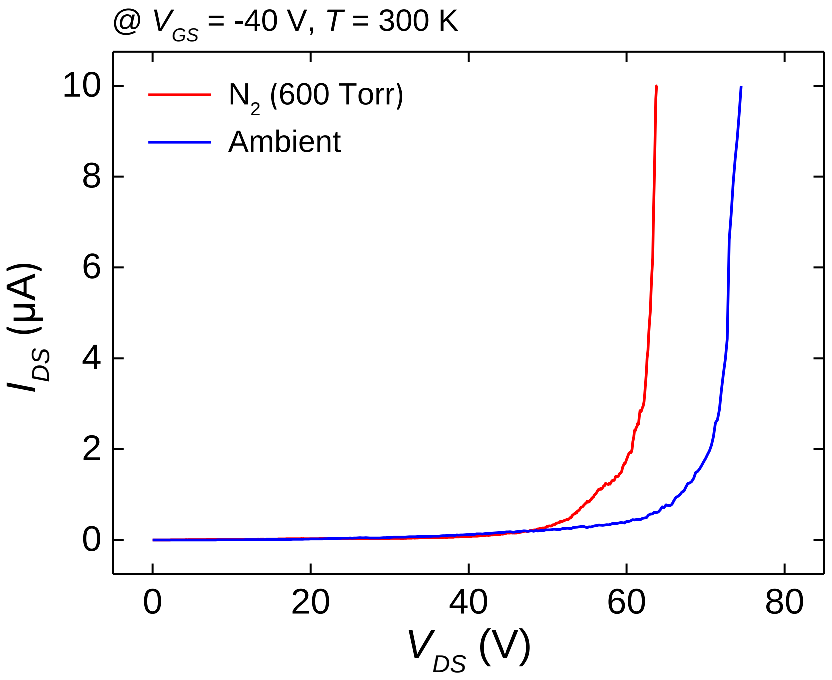

Figure S4. The electrical breakdown of the device in Figure 1d in the main manuscript measured in N_2 gas (red line) and ambient (blue line) environment.

4. Mechanism of an abrupt increase in I_{DS}

In general, there exist four possible mechanisms that can give rise to an abrupt increase in I_{DS} in FETs: drain-induced barrier lowering (DIBL), thermal breakdown, junction punch through, and avalanche carrier multiplication. First, DIBL is a representative short-channel effect that might exist in MoS_2 FETs. However, the electrostatic characteristics of monolayer MoS_2 gives rise to a negligible DIBL property even in ultra-short channel devices.[S6,S7] Second, it has been reported that there is a negligible amount of increase in the temperature of MoS_2 FETs fabricated on a SiO_2 substrate.[S6] Since hBN exhibits much better thermal conductivity (~420 W m^{-1} K^{-1}) than SiO_2 (~1.40 W m^{-1} K^{-1}),[S6,S8] the thermal effect resulting from Joule heating would be less significant in our device structures than MoS_2 FETs on a SiO_2 substrate. In this regard, thermal breakdown is also not a plausible mechanism to explain an abrupt increase in I_{DS}.

Third, we eliminate the possibility of junction punch through effect. We first outline a general mechanism of junction punch through effect in n-channel metal-oxide-semiconductor field-effect transistors (MOSFETs). Junction punch through effect occurs when the depletion region between the p-bulk and n+-source contact overlaps with that between the bulk and n+-drain contact, and thereby allowing source-drain current to flow through the overlapping depletion regions.[S9] Now, we represent the following reasons for neglecting the punch through effect as the origin of the abrupt increase in I_{DS} observed in this work. First, the lack of doped contact regions and atomically thin active channel of our CVD-grown MoS_2 monolayer FET devices do not allow the formation of such macroscopic overlap of depletion regions. Since the overlap of each depletion region arising from an n+-p junction lies at the heart of junction punch through, it is difficult to apply the mechanism of junction punch through in our devices. Second, the relationship between the electrical breakdown voltage (V_{EB}) and V_{GS} displayed in Figure 2e in the manuscript is not consistent with the prediction of junction punch through, even if we

assume that it can occur in atomically thin semiconductors. It should be noted that the width of a depletion region increases as a strong reverse bias is applied to p-n junction. Hence, as V_{GS} becomes more negative, a smaller value of V_{DS} is required for the formation of overlapping depletion regions. This implies that an abrupt increase in I_{DS} should start to be observed at a lower value of V_{DS} as more negative V_{GS} is applied. In contrast to this prediction, V_{EB} increased as V_{GS} became more negative (see Figure 2e in the manuscript). Thus, the increase in I_{DS} is attributed to avalanche multiplication rather than junction punch through. Fourth, we carried out further analyses to demonstrate that the photocurrent behavior does not follow the trend predicted by the junction punch through mechanism, as shown in Figure S5. According to the junction punch through theory, the current is proportional to the voltage squared,[S9] which implies that breakdown photocurrents should follow a power-law relation. In Figure S5, the breakdown photocurrent can be fitted better in an exponential increase rather than a power-law relation (see the dashed lines in Figure S5). This point is in contradiction with the outcome of the junction punch through theory. In this regard, avalanche multiplication is the most reasonable mechanism behind the abrupt increase in I_{DS}, as shown in literature.[S6]

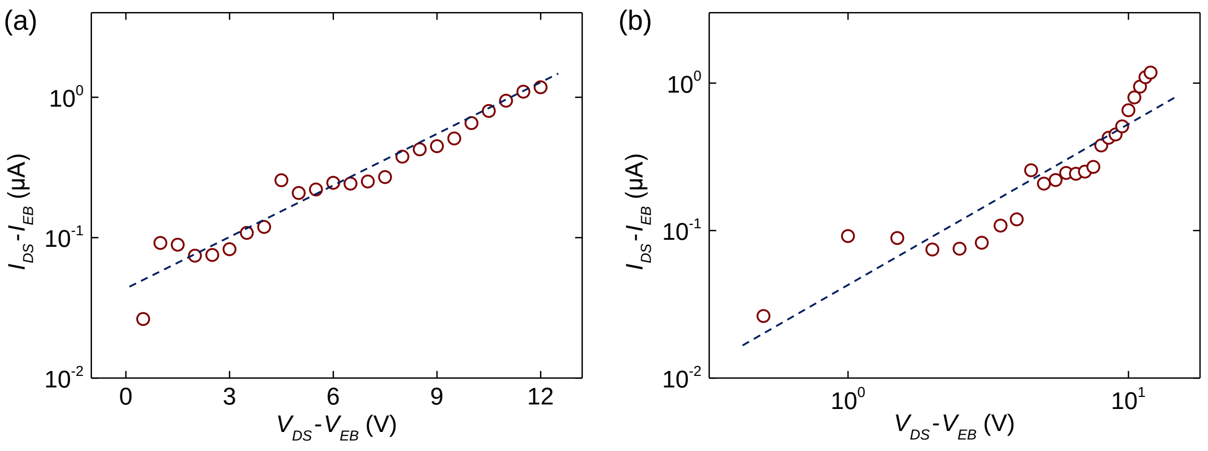

Figure S5. Breakdown of currents measured under laser irradiation demonstrated in (a) a semilogarithmic scale and (b) a log-log plot. The dashed lines in each figure shows the results of linear fitting. In each figure, I_{EB} = I_{DS} (V_{DS} = V_{EB}).

5. Absence of device degradation and calculation of impact ionization rates

To check whether the measurements of the electrical breakdown permanently damaged the devices, we measured the channel current by sweeping V_{DS} from 0 V to 30 V after each breakdown event up to four times. The corresponding outcome is shown in Figure S6a. Note that these results were obtained at the fixed V_{GS} of -20 V. As a result, no evidence for device failures and degradation was found.

The multiplication factor M can be defined as

$$M(V_{DS}) = \frac{I_{DS}(V_{DS})}{I_{sat}} \ , \qquad \text{(S2)}$$

where I_{sat} is the saturation current. To apply the multiplication factor in the calculation of the impact ionization rate α, the saturation current I_{sat} should be strictly established in the obtained electrical data. If the saturation was observed in I_{DS}-V_{DS} curves, $I_{sat} = I_{DS}(V_{DS} = V_{EB})$ where V_{EB} is the electrical breakdown voltage. According to Figure 2d in the main manuscript, it can be seen that the change in I_{DS} before the onset of the breakdown is negligible when V_{GS} = -30, -40 and -50 V. Therefore, we could calculate the impact ionization rates from the obtained electrical data when V_{GS} = -30, -40 and -50 V.

Figure S6b demonstrates the impact ionization rate α, which was calculated when V_{GS} = -30, -40 and -50 V by using the equation (3) in the main manuscript as a function of $1/E$. We assumed that the impact ionization rates of electrons and holes are comparable to each other. It is known that this assumption does not influence the final result critically.[S10] Details on this assumption require further studies. With this assumption, the relationship between the multiplication factor M and the impact ionization rate α is given by the equation (S3).[S10]

$$1 - \frac{1}{M} = \int_0^L \alpha(E)\, \mathrm{d}x \qquad \text{(S3)}$$

Here, L is the channel length so the region $0 \leq x \leq L$ corresponds to where the electric field E is applied. Note that E can be a function of x. In addition, we assume that the electric field in

the channel does not depend on the position x. Then, the x-dependence of α disappears and we obtain the equation $1 - \frac{1}{M} = L\alpha$, which is the same to the equation (3) in the main manuscript. It was shown that the equation (3) approximately holds in various semiconducting materials such as Si and Ge.[S10,S11]

The relationship between α and the electric field E is given by the equation (S4).[S10,S11]

$$\alpha = \alpha_{\infty}\exp(-\beta/E) \qquad \text{(S4)}$$

Based on the equation (S4), we performed fitting to the calculated values of impact ionization rates. The average R^2 value of the fitting shown in Figure S6b was ~0.91, which shows that the obtained relationship between α and $1/E$ fairly well agreed with the equation (S4).

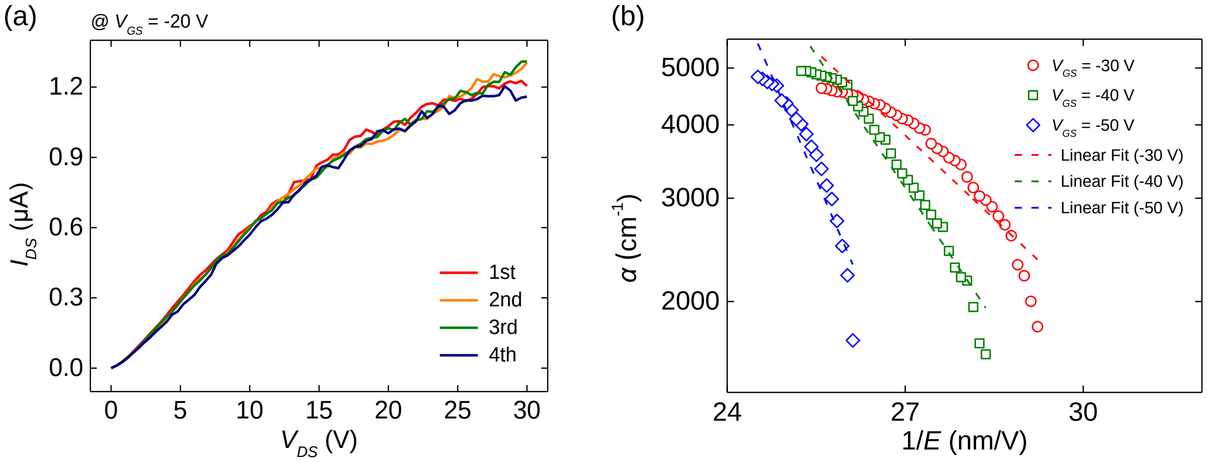

Figure S6. (a) I_{DS} versus V_{DS} after each breakdown measurement. The first (red) line was obtained after measuring the breakdown at V_{GS} = -10 V. The second (yellow) line was obtained after measuring the breakdown at V_{GS} = -20 V. The third (green) line was obtained after measuring the breakdown at V_{GS} = -30 V. The fourth (navy) line was obtained after measuring the breakdown at V_{GS} = -40 V. (b) Relationship between the ionization impact rates calculated when V_{GS} = -30, -40 and -50 V and applied electric field E. The dashed lines show the fitted lines based on the equation (S4). Note that α is represented in a logarithmic sale.

6. Electrical characteristics and breakdown of another MoS_2 FET

Figures S7a and S7b show the output and transfer curves of the MoS_2 FET shown in the inset of Figure S7a, respectively. The device shows an ohmic contact property. By using the equation (1) in the main manuscript, the field-effect mobility was estimated as ~14 cm^2 V^{-1}s^{-1}. Here, the channel length L = 3.0 μm, channel width W = 9.1 μm, and capacitance per unit area C_i = 10.5 nF cm^{-2} were used in the calculation. The thickness of the used hBN film was determined to be ~56 nm. We characterized the electrical properties of this device at high drain-source biases (V_{DS}), which is shown in Figure S7c. The electrical breakdown occurred at V_{DS} value of ~93 V. The corresponding critical electrical field was found to be ~0.33 MV cm^{-1}, which shows a great agreement with the values obtained in the main manuscript and the previously reported values.[S6] As the analyses in the main manuscript, we plotted 1-1/M as a function of V_{DS}/V_{EB}, where M is the multiplication factor and V_{EB} is the initial voltage of electrical breakdown. The value of m obtained by fitting was found to be ~7.1, which is in the range of what were obtained in the inset of Figure 2f in the main manuscript. As in the main manuscript, the fitting was performed just after the onset of the electrical breakdown.

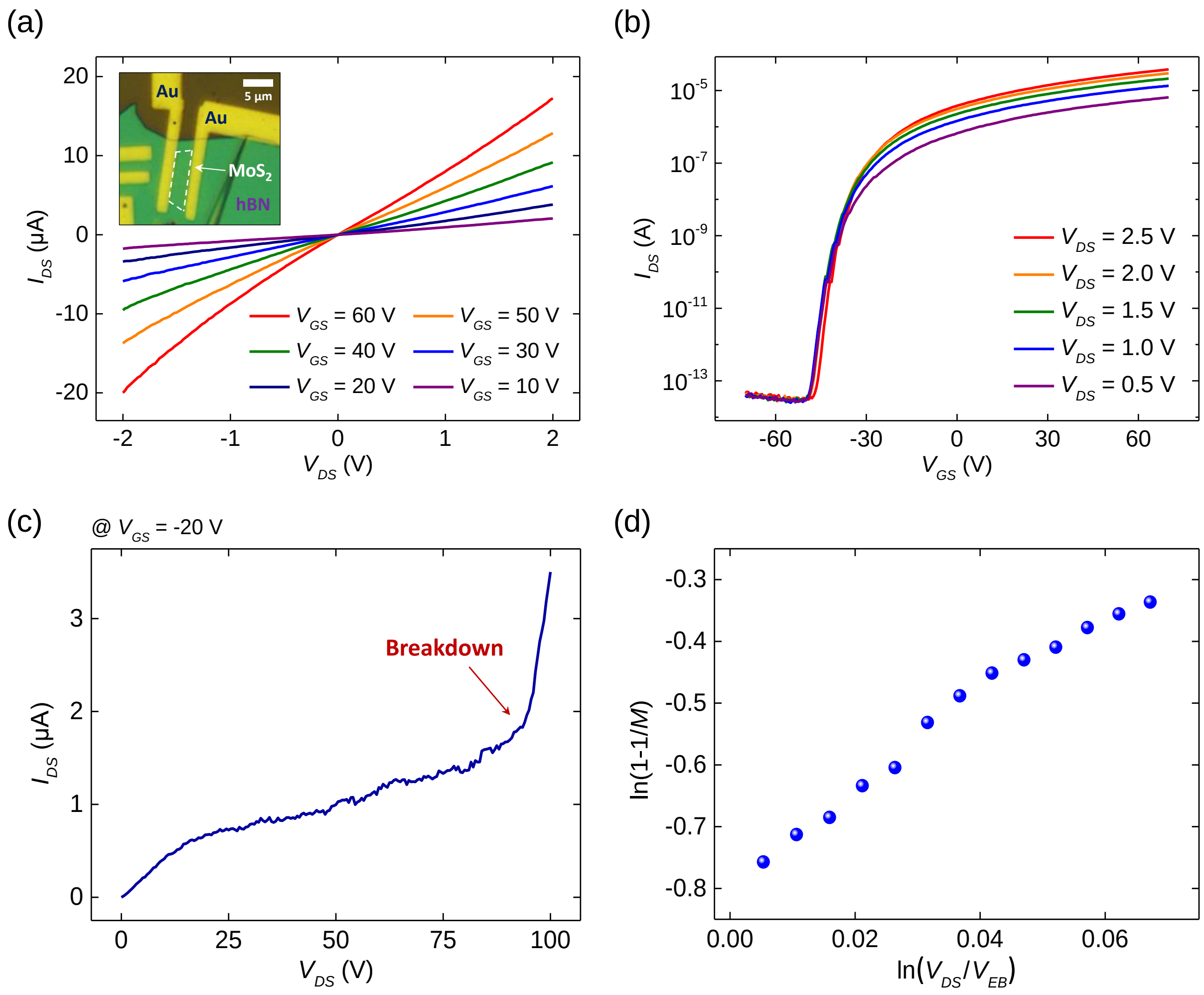

Figure S7. (a) The output curves of the device shown in the inset. (b) The transfer curves. (c) I_{DS}-V_{DS} curve measured at V_{GS} = -20 V exhibiting the avalanche breakdown. (d) Relationship between 1-1/M and V_{DS}/V_{EB} measured at V_{GS} = -20 V.

7. V_{DS}-dependent photoresponsivity and detectivity under high illumination intensity

Figure S8a shows the photoresponsivity and detectivity of the device shown in Figure 1d in the main manuscript measured under the laser irradiation with the intensity of 2.5 μW cm^{-2}. In the region B, the maximum values of photoresponsivity and detectivity were ~6.6 × 10^6 A W^{-1} and ~7.8 × 10^{15} Jones, respectively. In the region C, the maximum values of photoresponsivity and detectivity were ~8.8 × 10^6 A W^{-1} and ~7.7 × 10^{15} Jones, respectively. Note that all of these aforementioned values are higher than the maximum values of the previous reports (see Figure 4 in the main manuscript for the comparison to previously reported devices). Figure S8b demonstrates the relationship between 1-1/M and V_{DS}/V_{EB} corresponding to the I_{DS} measured in the dark and under the illumination (2.5 μW cm^{-2}). The values of m for the current measured in the dark and under illumination were ~24 and ~19, respectively. Similarly to other previous works, these values were obtained by fitting in the vicinity of ln(V_{DS}/V_{EB}) of ~0.05, namely just after the onset of the breakdown, which corresponds to the green region in Figure S8b. This result well agrees with the fact that the I_{irra} increased less abruptly after the onset of the breakdown compared to that measured in the dark (see Figure 3a in the main manuscript).

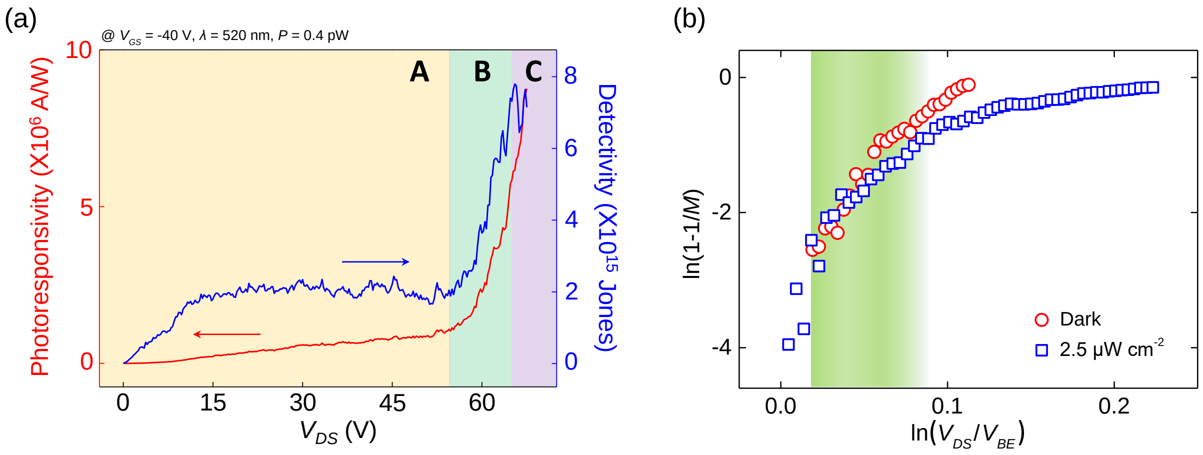

Figure S8. (a) Photoresponsivity and detectivity of the device shown in Figure 1d in the main manuscript under the laser intensity of 2.5 μW cm^{-2}. (b) Relationship between 1-1/M and V_{DS}/V_{EB} for the I_{DS} measured in the dark and under illumination (2.5 μW cm^{-2}). The green region denotes where fitting based on the equation (2) in the main manuscript was performed.

8. Illumination-time-dependent measurement of photo-initiated carrier multiplication

We characterized the breakdown of currents under irradiation with changing the laser illumination time to show that illumination time does not affect the devices through mechanisms except avalanche carrier multiplication such as photo-induced charge trapping. It is known that photo-induced charge trapping in 2D TMDCs can result in non-equilibrium characteristics with the timescale of a few to hundreds of seconds (see Section 14 and 15 in the Supporting Information for detailed discussions). It should be noted that in all the experiments in the main manuscript, the electrical breakdown was measured in full I_{DS}-V_{DS} scan. Since we set the value of V_{GS} such that the devices are in their off state, I_{DS} at a low-V_{DS} region was extremely low, which leads to quite a long time to measure I_{DS} as a function of V_{DS}. Hence, we measured I_{DS} at a partial range of V_{DS} for breakdown measurements. The corresponding results are shown in Figure S9. Owing to a smaller region of V_{DS} in the I_{DS}-V_{DS} scan (Figure S9), it took only ~5 s for a single sweep of V_{DS}, and therefore it was possible to measure the photoresponse after the illumination for a short period of time. As a result, no clear dependence of the photoresponse on the illumination time was found. During the experiments, the breakdown of dark currents did not change (see navy lines in Figure S9), which demonstrates that the device did not suffer from the change in its characteristics by continuous V_{DS} sweeping. Thus, we can safely say that the device condition was not significantly affected by illumination time, considering the timescale related to photo-induced charge trapping.

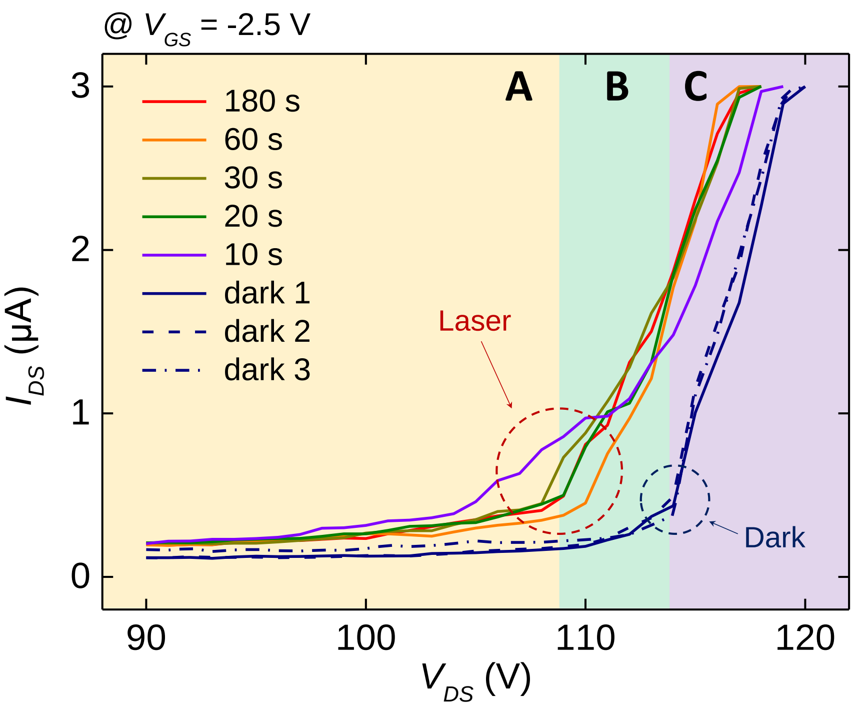

Figure S9. Breakdown of the dark current (navy, labelled as dark 1, 2 and 3) and photoresponse (other colors) after illuminating 520-nm laser for 10, 20, 30, 60 and 180 s. In all the measurements, a compliance current was 3 μA. Here, V_{GS} was set to be -2.5 V due to the low I_{DS} at this gate voltage.

9. Linearity of photocurrents

Our analyses of the relationship between the photocurrent and light intensity are based on the data shown in Figure S14a in Section 12 (see Section 12 for further details about the device). The dependence of the photocurrent I_{ph} on the power P of incident light for each V_{DS} is demonstrated in Figure S10a. To investigate the linearity of MoS_2 avalanche phototransistors, we fitted the data measured ad each V_{DS} by using the equation $I_{ph} \propto P^{\gamma_P}$. The dependence of the exponent γ_P on V_{DS} is illustrated in Figure S10b, which shows a general trend that γ_P decreases as V_{DS} increases. Since a condition γ_P =1 means a perfect linearity, Figure S10b denotes that the avalanche phototransistors exhibit stronger nonlinearity as V_{DS} increases and the device reaches high-sensitivity regimes. The decrease in γ_P can be explained by the enhanced impact of avalanche breakdown with an increase in V_{DS}. To put it concretely, photocurrents generated by the photoconductive effect are in general proportional to P, because $I_{ph,\mathrm{PC}} = \frac{W}{L} V_{DS} \Delta\sigma = \frac{W}{L} V_{DS} e\mu(\Delta n)$ where $\Delta n \propto P$ in the photoconductive effect (see Section 15 for details about the photoconductive effect). In contrast, the number of charge carriers generated by avalanche breakdown is expected to be more or less independent of P, since photons do not play a role when high-energy carriers create new electron-hole pairs via collisions with the lattice. Hence, γ_P deviates from 1 by a larger amount as V_{DS} increases and the avalanche carrier multiplication becomes more significant. It is known that large photogating effects can also make γ_P deviate from 1,[S12] but we believe that this effect is negligible in this case since V_{GS}, which alters the photogating, was kept constant during the whole measurements. In addition, we showed the dependence of photoresponsivity R on P in Figure S10c and S10d, and fitted the obtained data by using the equation $R \propto P^{-\gamma_R}$. According to Figure S10d, stronger dependence of R on P (i.e. larger γ_R) was observed as V_{DS} increased.

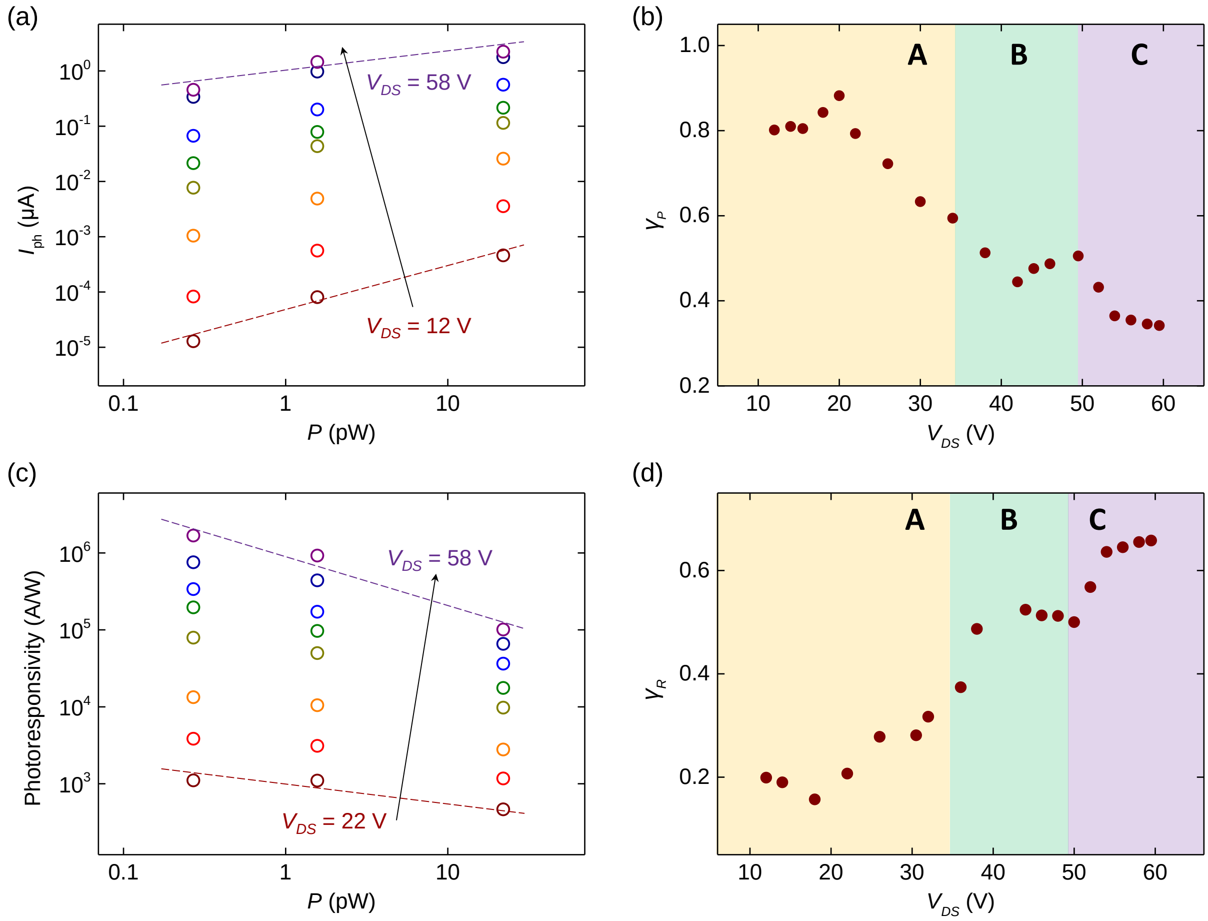

Figure S10. (a) Dependence of the photocurrent I_{ph} on P at V_{DS} of 12 V, 18 V, 26 V, 34 V, 38 V, 44 V, 54 V, and 58 V. The dotted lines indicate fitted results, where a decrease in γ_P is evident. (b) Dependence of γ_P on V_{DS}. (c) Dependence of the photoresponsivity R on P at V_{DS} of 22 V, 26 V, 30 V, 38 V, 42 V, 46 V, 52 V, and 58 V. The dotted lines indicate fitted results, where an increase in γ_R is evident. (d) Dependence of γ_R on V_{DS}.

10. Photo-initiated carrier multiplication under illumination with different wavelengths

We measured the response of one of our devices (different from the device shown in the main manuscript) to the incident light with the wavelength of 405, 520 and 658 nm. The corresponding results are demonstrated in Figure S11. When performing the experiments, we fixed the gate-source voltage to be -10 V so that the dark current was sufficiently low. We also did our best to use the laser beam with the same intensity. For all the values of wavelength used here, regions A, B and C that are explained in the main manuscript were clearly visible (see Figures S11a, S11c and S11e). This indicates that ultrasensitive photodetection based on avalanche carrier multiplication was possible in a broad range of visible light spectrum (from 405 to 658 nm). This observation is in agreement with the fact that the photon energy (~1.88 eV) of 658-nm light is larger than the optical bandgap (~1.84 eV) of the synthesized monolayer MoS_2 used in this work (see Figure 1c in the manuscript). Moreover, it should be noted that the photoresponsivity of the device measured in Figures S11a, S11c and S11e is in quite good agreement with that obtained in the corresponding transfer curves (Figures S11b, S11d and S11f, respectively), which denotes that the breakdown measurements were appropriately performed.

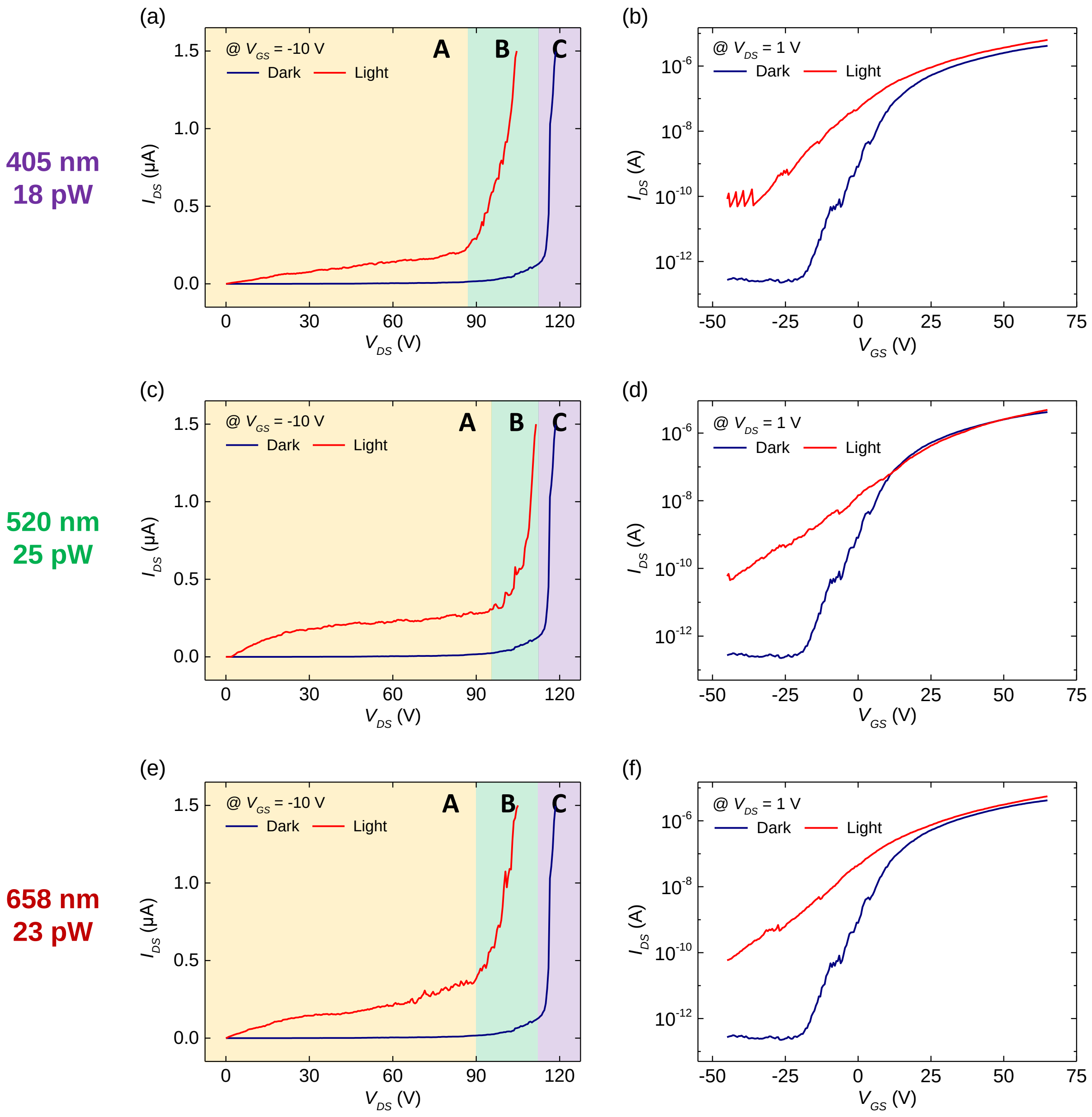

Figure S11. (a, c, e) Breakdown measurements in the dark and under (a) 405-nm, (c) 520-nm, and (e) 658-nm irradiation. (b, d, f) Transfer curves in the dark and under (b) 405-nm, (d) 520-nm, and (f) 658-nm irradiation. In all the breakdown measurements, a compliance current was 1.5 μA.

11. Comparison of field-effect mobility

In Table S1, we compared the field-effect mobility of our device shown in Figure 1d in the manuscript with previously reported monolayer MoS_2 FETs. It should be noted that the field-effect mobility of 2D materials tends to increase as the thickness increases,[S13] and thus we did not compared our device to multi-layered MoS_2 FETs. Some devices in literature demonstrated better transport characteristics and higher mobility, according to Table S1. This means that it is possible to improve field-effect mobility by using strategies already reported, which is beyond the scope of this work. Under low electric fields, the drift velocity of charge carriers is proportional to the field-effect mobility. Thus, the photoresponsivity R is roughly proportional to the mobility μ as well; $R \propto I_{ph} \propto ne\mu$ where I_{ph} is the photocurrent, n is the concentration of photogenerated charge carriers, and e is the elementary charge. This implies that further improvements on electrical properties should enable the realization of more sensitive MoS_2 avalanche phototransistors.

Table S1. Field-effect mobility of monolayer MoS_2 FETs measured by two-point-probe method.

Reference	Mobility (cm^2 V^{-1} s^{-1})	Reference	Mobility (cm^2 V^{-1} s^{-1})
Our work	54	S19	73
S14	60	S20	22
S15	42.3	S21	24
S16	60	S22	19
S17	17	S23	84
S18	45.1	S24	35

12. MoS_2 avalanche phototransistors with Pd contact

In this section, we demonstrated MoS_2 avalanche phototransistors with Pd contact, which are expected to have higher contact barrier height due to the work function of Pd.[S2,S4]

A. Electrical characteristics of the MoS_2 FET with Pd contact

Figure S12a shows the optical image of a MoS_2 FET with Pd contact fabricated on a hBN flake. The thickness of Pd metals was 60 nm. Figure S12b demonstrates the AFM image of the hBN flake in Figure S12a. The thickness of the hBN flake was determined to be ~12 nm. Figures S12c and S12d show the output and transfer curves of the device, respectively. The device

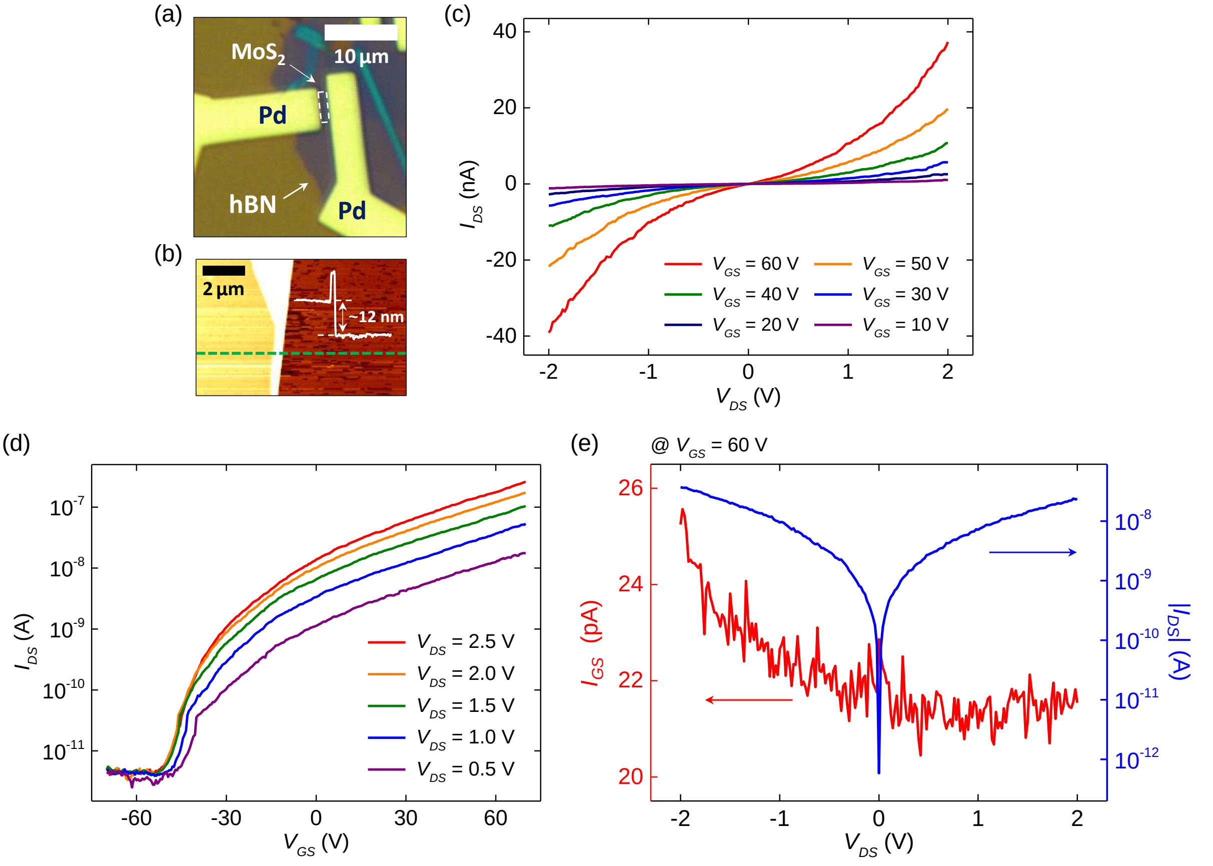

Figure S12. (a) Optical image of a MoS_2 FET with Pd contact. (b) AFM image of the hBN flake shown in (a) with its topographic cross-sectional profile along the green dashed line. (c) The output curves and (d) transfer curves of the device. (e) V_{DS}-dependent gate-source current (I_{GS}) and the absolute value of I_{DS} measured at V_{GS} = 60 V.

exhibited a non-ohmic-contact property even at high V_{GS}. The field-effect mobility of the FET was estimated to be ~9.4 × 10^{-2} cm^2 V^{-1} s^{-1} by using the equation (1) in the main manuscript with the channel length L=1.0 μm, channel width W=4.9 μm, and capacitance per unit area C_i = 12.2 nF cm^{-2}. Figure S12e shows that the gate leakage of this device is negligible.

B. Electrical breakdown of the MoS_2 FET with Pd contact

Figure S13a shows the electrical breakdown for the device shown in Figure S12a. Unlike the Au-contact device, the Pd-contact FET device did not show any noticeable V_{GS} dependence of the breakdown. This point is represented in Figure S13b. Unlike Figure 2e in the main manuscript, no noticeable pattern of V_{EB} and $\Delta I_{DS}/\Delta V_{DS}$ determined by V_{GS} was observed. This behavior can be attributed to an overwhelmingly high contact barrier between MoS_2 and Pd.[S4] Thus, the change in the barrier by the modulation of V_{GS} does not play an important role, and the dependence of V_{EB} and $\Delta I_{DS}/\Delta V_{DS}$ on V_{GS} could not be observed.

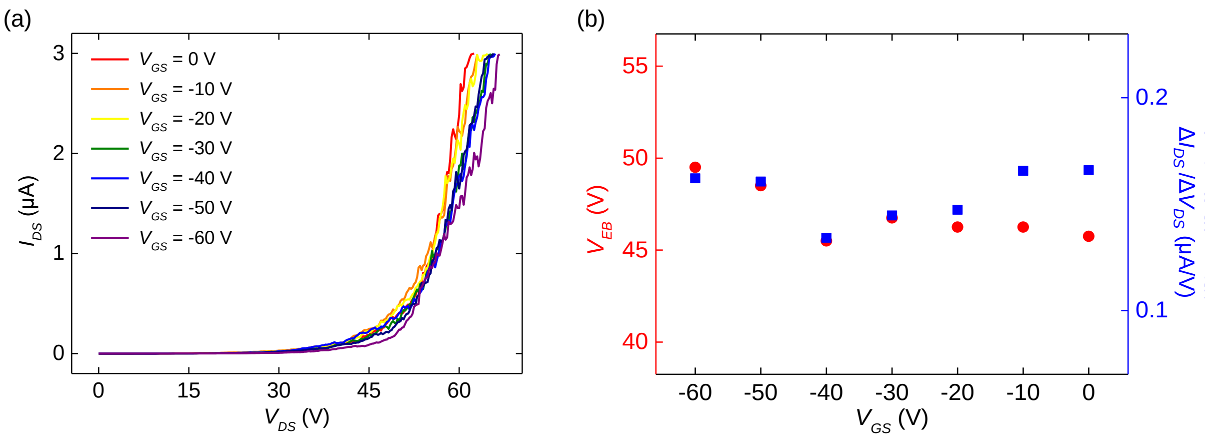

Figure S13. (a) V_{GS}-dependent I_{DS}-V_{DS} curves at high drain-source biases. (b) V_{GS}-dependent values of V_{EB} and $\Delta I_{DS}/\Delta V_{DS}$.

C. Photoresponse of the MoS_2 FET with Pd contact under various laser intensities

Figure S14a shows the photoresponse of the Pd-contact MoS_2 FET shown in Figure S11a. The photoresponse was measured at V_{GS} = -60 V and three different values of laser intensity. As other Au-contact MoS_2 FETs (see Figure 3 in the main manuscript and Figure S11 in the Supporting Information), the values of V_{DS} could also be classified into three regions. In the region A (V_{DS} < 34 V), no electrical breakdown was observed when I_{DS} was measured both in the dark and under illumination. In the region B (34 V < V_{DS} < 49 V), only the breakdown was observed when the device was irradiated by the laser. In the region C (49 V < V_{DS}), both currents measured in the dark and under illumination exhibited the electrical breakdown. In case of this device, the length of the region B was ~15 V in voltage, which corresponds to ~0.15 MV cm^{-1} in the unit of electric fields. This value is about 2.4 times larger than that of the Au-contact FET.

Figures S14b−S14d respectively show the dependence of photoresponsivity and detectivity on V_{DS} under different laser intensities. First, note that the values of the photoresponsivity decreased as the light intensity increased. Second, a decrease in the detectivity along with increasing V_{DS} could be observed in the region C. This was due to an abrupt increase in the dark current in the region C. We stress that the photoresponsivity generally increased with V_{DS}, unlike the detectivity. In case of the Pd-contact MoS_2 FET, the maximum values of photoresponsivity, detectivity and EQE in the region B were found to be ~4.6×10^5 A W^{-1}, ~5.6×10^{14} Jones and ~1.1×10^8 %, respectively. Also, the maximum values of photoresponsivity, detectivity and EQE in the region C were ~3.8×10^6 A W^{-1}, ~4.1×10^{14} Jones and ~9.1×10^8 %, respectively. Again, we could confirm the enormous power of the avalanche breakdown in that we could greatly enhance the performance of the device that showed much worse photoresponsivity at low drain-source bias regions (11.3 A W^{-1} at V_{GS} = -60 V, V_{DS} = 8 V and P = 1.57 pW) in comparison to previously reported MoS_2 FETs.[S25,S26]

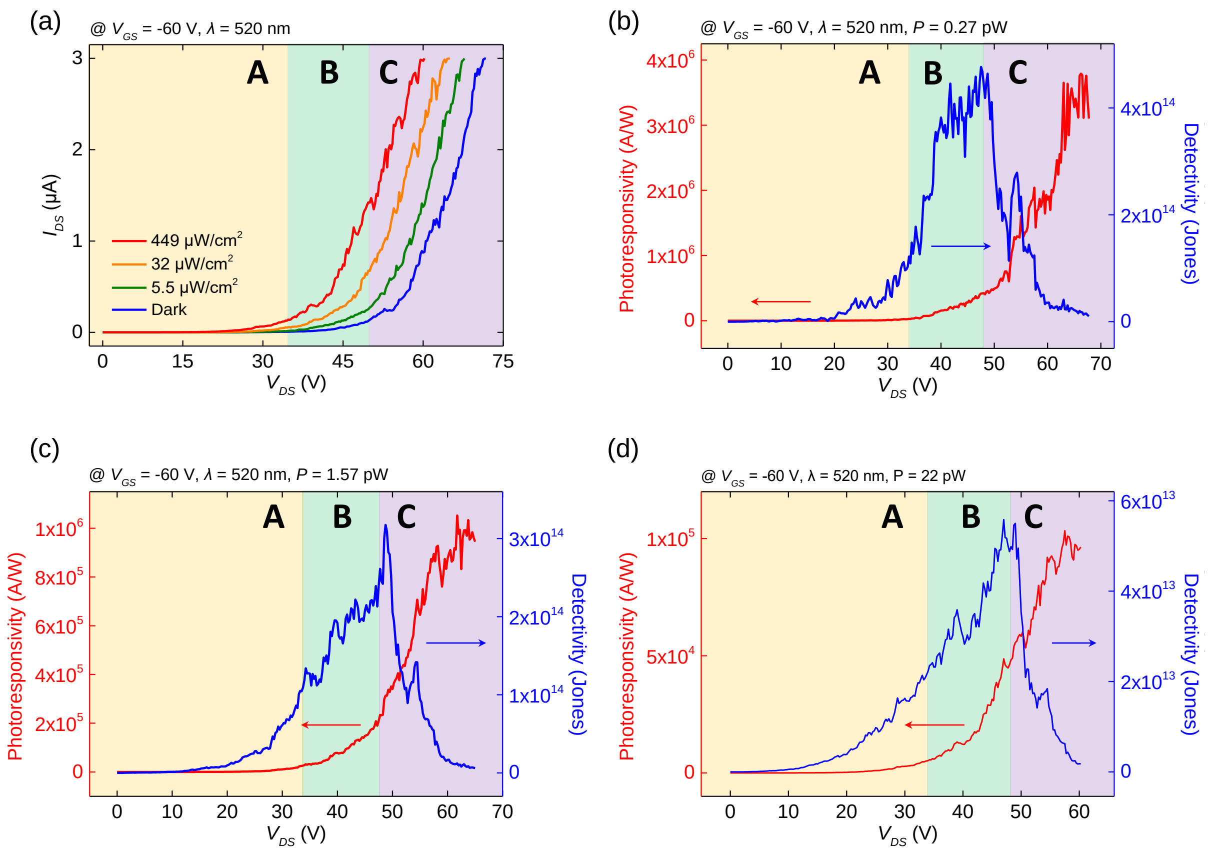

Figure S14. (a) I_{DS} measured in the dark and under the laser irradiation with different intensity. (b) Photoresponsivity and detectivity values calculated under the irradiation with the intensity of 5.5 μW cm^{-2} versus V_{DS}. (c) Photoresponsivity and detectivity values calculated under the irradiation with the intensity of 32 μW cm^{-2} versus V_{DS}. (d) Photoresponsivity and detectivity values calculated under the irradiation with the intensity of 449 μW cm^{-2} versus V_{DS}.

D. Physical explanation of Pd-contact MoS_2 avalanche phototransistors

The energy band diagrams in Figure 3c, d, e in the main manuscript implies that the voltage window of the region B, which is essential for the stable operation of MoS_2 avalanche phototransistors, can be controlled by modulating the contact barrier height. We stress that MoS_2 FETs with Pd contact shown in this section materializes this possibility. It is well known that a contact barrier between MoS_2 and Pd is higher than that between MoS_2 and Au.[S2,S4] Accordingly, the Pd-contact MoS_2 FET showed a typical *n*-type and non-ohmic-contact behavior (see Figure S12). Unlike the Au-contact MoS_2 FET, the values of V_{EB} obtained in the

Pd-contact device did not show a strong V_{GS} dependence (see Figure S13) because the contact barrier in the Pd-contact device is already too high for charge carriers to overcome regardless of the value of V_{GS}.[S4] Furthermore, the critical electric field for dark currents (i.e., $E_{CR,\mathrm{dark}}$) of this device was estimated as ~0.46 MV cm^{-1} at all the V_{GS} values which is larger than that of the Au-contact device (between ~0.26 MV cm^{-1} and ~0.37 MV cm^{-1} in the V_{GS} range from -20 V to -50 V). This is a supporting evidence for our device operation model outlined in Figure 3c, d, e as the higher contact barrier in the Pd-contact device delays the V_{DS} onset of the electrical breakdown in the dark condition. According to photoresponse of the Pd-contact device (see Figure S14), the region A corresponded to the values of V_{DS} smaller than ~34 V. The critical electric field for I_{irra} (*i.e.*, $E_{CR,\mathrm{irra}}$) was ~0.34 MV cm^{-1} for the Pd-contact device, which is comparable to ~0.29 MV cm^{-1} for the Au-contact device. Considering that most of the V_{DS} is dissipated in the channel (see Figure S15 and related discussions in Section 13), the minute difference between the $E_{CR,\mathrm{irra}}$ of Au- and Pd-contact devices indicates that the breakdown under the light condition is hardly influenced by the contact barrier height. This also supports that the breakdown of our devices in the region B was triggered by the photogenerated carriers rather than the electrically injected carriers. Moreover, the regimes 34 V < V_{DS} < 49 V and 49 V < V_{DS} corresponded to the region B and C, respectively. In case of the Pd-contact device, the voltage window of the region B was ~15 V, which corresponds to ~0.15 MV cm^{-1}. This value is ~2.4 times larger than that of the Au-contact device, which also supports the validity of the energy band diagrams that we proposed above.

13. Contact resistance of the MoS_2 FET with Au and Pd contact

The contact resistance of FETs was extracted by using the Y-function method.[S27] This method has been widely used to estimate the contact resistance for diverse materials.[S28-S30] The channel current (I_{DS}) in a linear region can be expressed as following.

$$I_{DS} = \frac{\mu_0}{1+\theta_{ch}(V_{GS}-V_{th})} C_i \frac{W}{L} (V_{GS}-V_{th})(V_{DS}-I_{DS}R_C)$$

$$= \frac{\mu_0}{1+\theta(V_{GS}-V_{th})} C_i \frac{W}{L} (V_{GS}-V_{th}) V_{DS}$$

Here, μ_0, θ_{ch}, C_i, L, W, R_C, V_{th}, and θ are respectively the intrinsic mobility in the linear region, the mobility attenuation factor from the channel, the capacitance between the channel and the gate per unit area, the channel length, the channel width, the contact resistance, the threshold voltage and the mobility attenuation factor from both the channel and contact regions. The Y-function is defined as

$$Y = \frac{I_{DS}}{\sqrt{g_m}} = \frac{\mu_0}{\sqrt{I_{DS}(V_{GS}-V_{th})/[1+\theta(V_{GS}-V_{th})]}} = \sqrt{\mu_0 C_i V_{DS} \frac{W}{L}} (V_{GS}-V_{th}) \ ,$$

where $g_m = \partial I_{DS}/\partial V_{GS}$ is transconductance. Here, the attenuation factor θ can be expressed as $\theta = \theta_{ch} + \theta_c = \theta_{ch} + \mu_0 C_i R_C \frac{W}{L}$ where θ_c is the mobility attenuation factor from the contact regions. Therefore, the contact resistance can be obtained as $R_C = \frac{S_2}{S_1} V_{DS}$ where S_1 and S_2 are the derivative values of the Y-function and transconductance by V_{GS}.

Figures S15a and S15b demonstrate the Y-function and transconductance data of the Au- and Pd-contact FET, respectively. For the Au-contact device, the obtained values of S_1 and S_2 were 4.00×10^{-4} A V^{-1} $S^{-0.5}$ and 20.8 V^{-1} $S^{-0.5}$, respectively. For the Pd-contact device, the estimated values of S_1 and S_2 were 1.46×10^{-5} A V^{-1} $S^{-0.5}$ and 6.88×10^{3} V^{-1} $S^{-0.5}$, respectively. We note that these values were obtained at V_{DS} = 1 V. As a result, the contact resistance values of the Au- and Pd- contact device were determined to be 51.9 kΩ and 47.1 MΩ, respectively. These values are consistent with the previously reported values.[S4]

We next determined the channel resistance of the devices in the off state. Since the FETs are in the off state, the Fermi level is at the middle of the bandgap of MoS_2. It is known that the resistivity of monolayer MoS_2 without any doping (i.e., the Fermi level is at the middle of the bandgap) is larger than 5000 Ω cm.[S31] Thus, the channel resistance values of the Au- and Pd-contact device are larger than 13.4 GΩ and 12.1 GΩ, respectively. These values are at least few orders of magnitude larger than the contact resistance. This means that the applied drain-source bias was largely applied to the channel when performing the electrical breakdown measurements in the off state.

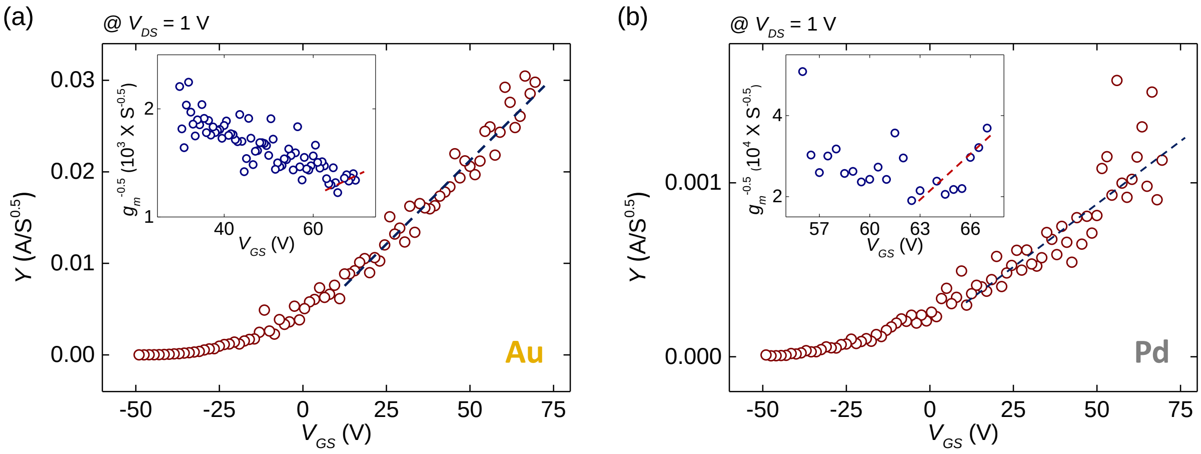

Figure S15. Calculated Y-function and transconductance (inset) of (a) Au- and (b) Pd-contact FET shown in Figure 1d in the main manuscript and Figure S12a, respectively, at V_{DS} = 1 V. Dashed lines indicate the fitted regions in each curve to obtain S_1 and S_2.

14. Characterization and comparison of photoswitching dynamics

Figure S16 shows the photoswitching dynamics for devices used in our work. Here, the photoswitching behaviour was characterized by illuminating a device with a continuous wave laser and measuring I_{DS} by ammeter. All of the devices exhibited stable photoswitching characteristics. It took a few to few tens of seconds for the photocurrent stabilization when the laser irradiation was turned on and few seconds for the disappearance of the photocurrent after turning off the laser. To be specific, for the device 1, 2 and 3 (see Figure S16a, S16b and S16c, respectively), the rise time was 27 s, 4.1 s and 4.8 s and the decay time was 1.1 s, 2.9 s and 0.2 s, respectively. Similarly to previous works, the rise time was defined as the period of time required for the photocurrent to change from 10% to 90% of its maximum value, and the decay time was defined as the time at which the photocurrent decreases to 1/e of its initial value.

Table S2 shows the comparison of device performance parameters (photoresponsivity, rise time, decay time). According to Table S2, the response times of our devices were comparable or shorter than many devices previously reported in literature. However, we could not obtain microsecond-range photoswitching response times in our devices. We believe that it will be possible to further enhance the photoswitching characteristics by reducing the photogating effect (see Section 15 for further details), which significantly affects the photoswitching dynamics of 2D-TMDC-based phototransistors.[S32]

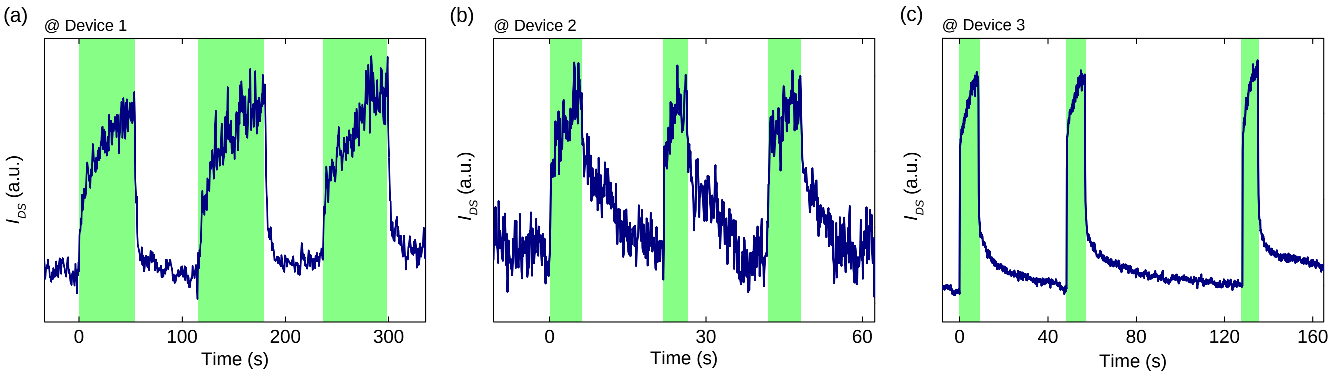

Figure S16. Photoswitching dynamics of three devices. The green boxes indicate when 520-nm-wavelength laser was irradiated into the device.

Table S2. Comparison of a channel material, photoresponsivity, rise and decay time with the characterization method. Reference numbers without "S" are those cited in the main manuscript.

Ref	Channel Material	Photoresponsivity (A/W)	Rise Time (s)	Decay Time (s)
Ours	MoS_2	3.4×10^7	27	1.2
18	MoS_2	2×10^4	NA	3
36	MoS_2	880	4	9
37	MoS_2	2200	25	500
38	MoS_2	$\approx 10^4$	400	120
S33	MoS_2	1	NA	≈67
S34	MoS_2	< 34	13	3.6
S35	MoS_2	< 0.02	≈3	≈4
44	$MoSe_2$	97.1	7	≈1
46	WS_2	61	0.002	0.002
49	WS_2	0.7	25	≈1
50	WSe_2	0.6	8×10^{-6}	8×10^{-6}
60	WSe_2	25	92	58
17	MoS_2/Perovskite	$\approx 10^4$	7	5
52	MoS_2 / Perovskite	$\approx 10^4$	0.6	0.3
54	MoS_2 / ZnCdSe	5000	0.3	1.2
S36	MoS_2 / Graphene	≈9	≈6	≈4
47	In/Graphene-WS_2-Graphene	2600	47×10^{-6}	47×10^{-6}
58	Reduced Graphene Oxide/MoS_2	21.8	2.8×10^{-6}	47×10^{-6}
59	MoS_2/Ferroelectric	2570	0.002	0.002

15. Photogating effect of MoS_2 FETs

Among various mechanisms that explain the generation of photocurrent in photodetectors based on 2D materials, photoconductive and photogating effects play the most significant role when the drain-source bias is applied to the devices.[S37] According to the photoconductive effect, free electrons and holes are created by the incident light and an increase in the number of free charge carriers results in the photocurrent. According to the photogating effect, photogenerated carriers are trapped in states arising from structural defects, disorders, or imperfect interface. The trapped charges provide additional gate fields, which gives rise to a shift in the threshold voltage. This indicates that the threshold voltage shift is one of the distinctive characteristics of the photogating effect. In overall, the photocurrent (I_{ph}) can be expressed as $I_{ph} = I_{ph,\mathrm{PC}} + I_{ph,\mathrm{PG}} = \frac{W}{L} V_{DS} \Delta\sigma + g_m |\Delta V_{th}|$, Here, $I_{ph,\mathrm{PC}}$ and $I_{ph,\mathrm{PG}}$ are the photocurrent due to the photoconductive effect and photogating effect, respectively, W is device channel width, L is channel length, V_{DS} is drain-source voltage, g_m is differential transconductance, $\Delta\sigma = (\Delta n)e\mu$ is the change in the photoconductivity by the photogenerated charge carriers, and ΔV_{th} is the threshold voltage shift by the photogating effect.[S33,S38] Figures S17a and S17b show the output curves and transfer curves of the device shown in the inset of Figure S17a, respectively. The device showed an ohmic-contact property and the on/off ratio of higher than 10^8. According to Figure S17c, the threshold voltage of the device shifted in the negative-V_{GS} direction as the laser intensity increased. As mentioned earlier, this is a direct signature of the photogating effect. Our results demonstrate that MoS_2 FETs fabricated on hBN flakes exhibit a fairly strong photogating effect. It has also been reported that deep trap sites in hBN may strengthen the photogating effect.[S39]

In addition to photoresponsivity, the photogating effect has a significant impact on the photoswitching response times. Concerning a rise time, photogenerated carriers start to be trapped in empty trap sites when laser is irradiated to a device. During this process, V_{GS}

continuously changes and this leads to additional charge injection from the drain and source to the channel. This additional charge injection gives rise to a long rise time because it takes a certain amount of time for I_{DS} to reach a steady state. Since the photogating effect dominates in most 2D phototransistors, they can exhibit a comparatively long rise time up to hundreds of seconds as shown in Table S2. Concerning a decay time, photogenerated electrons and holes remain to be trapped in trap sites for a certain period of time even after laser illumination is turned off. Due to the trapped photogenerated carriers, the channel is not electrically neutral after turning off the irradiation, thereby leading to additional charge injection from the drain and source. Thus, the photocurrent does not immeadiately vanish but decreases within a certain amount of time. This can cause a long decay time in typical 2D phototransistors up to hundreds of seconds (see Table S2). This implies that further optimizations on the quality of MoS_2 films and MoS_2/dielectric interfaces can decrease the number of trap sites, which will make it possible to enhance the photoswitching characteristics of 2D phototransistors. For example, a fabrication of graphite-gated hBN-encapsulated devices that allow ultraclean interfaces and low trap densities can make the photoswitching response of MoS_2 avalanche phototransistors much faster.[S40]

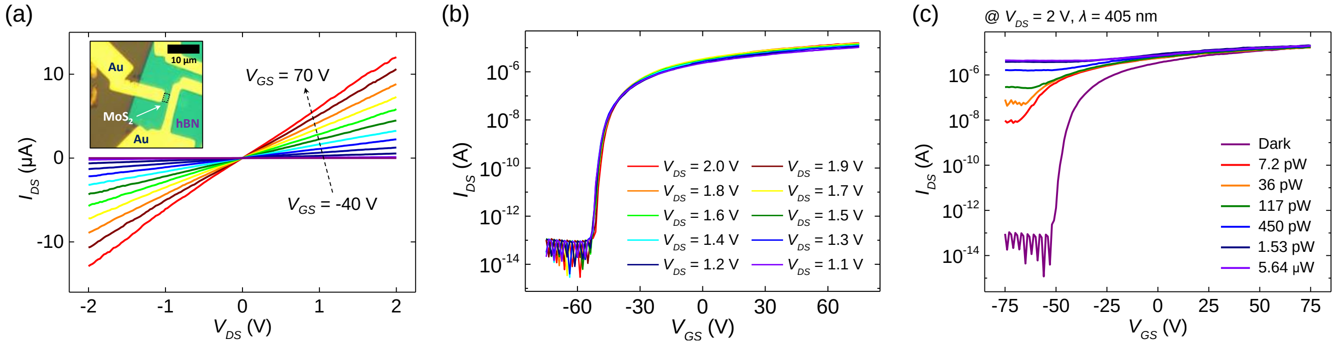

Figure S17. (a) The output curves of the device shown in the inset. (b) The transfer curves. (c) The transfer curves measured in the dark and under laser irradiation with different intensities. The wavelength of the laser beam was 405 nm.

References

[S1] R. V. Gorbachev, I. Riaz, R. R. Nair, R. Jalil, L. Britnell, B. D. Belle, E. W. Hill, K. S. Novoselov, K. Watanabe, T. Taniguchi, A. K. Geim, P. Blake, *Small* **2011**, 7, 465.

[S2] A. Allain, J. Kang, K. Banergee, A. Kis, *Nat. Mater.* **2015**, 14, 1195.

[S3] J. Kwon, C. J. Delker, D. B. Janes, C. T. Harris, S. R. Das, *Phys. Status Solidi A* **2020**, 217, 1900880.

[S4] N. Kaushik, A. Nipane, F. Basheer, S. Dubey, S. Grover, M. M. Deshmukh, S. Lodha, *Appl. Phys. Lett.* **2014**, 105, 113505.

[S5] S. Walia, S. Balendhran, Y. Wang, R. A. Kadir, A. S. Zoolfakar, P. Atkin, J. Z. Ou, S. Sriram, K. Kalantar-zadeh, M. Bhaskaran, *Appl. Phys. Lett.* **2013**, 103, 232105.

[S6] J. Pak, Y. Jang, J. Byun, K. Cho, T.-Y. Kim, J.-K. Kim, B. Y. Choi, J. Shin, Y. Hong, S. Chung, T. Lee, *ACS Nano* **2018**, 12, 7109.

[S7] Y. Yoon, K. Ganapathi, S. Salahuddin, *Nano Lett.* **2011**, 11, 3768.

[S8] P. Jiang, X. Qian, R. Yang, L. Lindsay, *Phys. Rev. Mater.* **2018**, 2, 064005.

[S9] R. F. Pierret, Semiconductor Device Fundamentals, Addison Wesley, **1996**.

[S10] K. G. McKay, *Phys. Rev.* **1954**, 94, 877.

[S11] K. G. McKay, K. B. McAfee, *Phys. Rev.* **1953**, 91, 1079.

[S12] J.-Y. Wu, Y. T. Chun, S. Li, T. Zhang, J. Wang, P. K. Shrestha, D. Chu, *Adv. Mater.* **2018**, 30, 1705880.

[S13] X. Cui, G.-H. Lee, Y. D. Kim, G. Arefe, P. Y. Huang, C.-H. Lee, D. A. Chenet, X. Zhang, L. Wang, F. Ye, F. Pizzocchero, B. S. Jessen, K. Watanabe, T. Taniguchi, D. A. Muller, T. Low, P. Kim, J. Hone, *Nat. Nanotechnol.* **2015**, 10, 534.

[S14] D. Jariwala, V. K. Sangwan, D. J. Late, J. E. Johns, V. P. Dravid, T. J. Marks, L. J. Lauhon, M. C. Hersam, *Appl. Phys. Lett.* **2013**, 102, 173107.

[S15] P.-Z. Shao, H.-M. Zhao, H.-W. Cao, X.-F. Wang, Y. Pang, Y.-X. Li, N.-Q. Deng, J. Zhang, G.-Y. Zhang, Y. Yang, S. Zhang, T.-L. Ren, *Appl. Phys. Lett.* **2016**, 108, 203105.

[S16] N. Huo, Y. Yang, Y.-N. Wu, X.-G. Zhang, S. T. Pantelides, G. Konstantatos, *Nanoscale* **2018**, 10, 15071.

[S17] W. Wu, D. De, S.-C. Chang, Y. Wang, H. Peng, J. Bao, S.-S. Pei, *Appl. Phys. Lett.* **2013**, 102, 142106.

[S18] Y. Patil, J. Kim, K. Agrawal, T. Park, J. Yi, N. Aoki, K. Watanabe, T. Taniguchi, G.-H. Kim, *Nanotechnology* **2021**, 32, 325603.

[S19] J. Wang, Q. Yao, C.-W. Huang, X. Zou, L. Liao, S. Chen, Z. Fan, K. Zhang, W. Wu, X. Xiao, C. Jiang, W.-W. Wu, *Adv. Mater.* **2016**, 28, 8302.

[S20] J. Zheng, X. Yan, Z. Lu, H. Qiu, G. Xu, X. Zhou, P. Wang, X. Pan, K. Liu, L. Jiao, *Adv. Mater.* **2017**, 29, 1604540.

[S21] Z. Zhang, X. Xu, J. Song, Q. Gao, S. Li, Q. Hu, X. Li, Y. Wu, *Appl. Phys. Lett.* **2018**, 113, 202103.

[S22] J.-K. Kim, Y. Song, T.-Y. Kim, K. Cho, J. Pak, B. Y. Choi, J. Shin, S. Chung, T. Lee, *Nanotechnology* **2017**, 28, 47LT01.

[S23] G. Wu, X. Wang, Y. Chen, Z. Wang, H. Shen, T. Lin, W. Hu, J. Wang, S. Zhang, X. Meng, J. Chu, *Nanotechnology* **2018**, 29, 485204.

[S24] S.-S. Chee, D. Seo, H. Kim, H. Jang, S. Lee, S. P. Moon, K. H. Lee, S. W. Kim, H. Choi, M.-H. Ham, *Adv. Mater.* **2019**, 31, 1804422.

[S25] O. Lopez-Sanchez, D. Lembke, M. Kayci, A. Radenovic, A. Kis, *Nat. Nanotechnol.* **2013**, 8, 497.

[S26] W. Zhang, J.-K. Huang, C.-H. Chen, Y.-H. Chang, Y.-J. Cheng, L.-J. Li, *Adv. Mater.* **2013**, 25, 3456.

[S27] G. Ghibaudo, *Electron. Lett.* **1988**, 24, 543.

[S28] S. J. Choi, P. Bennett, K. Takei, C. Wang, C. C. Lo, A. Javey, J. Boker, *ACS Nano* **2013**, 3, 798.

[S29] Y. Kim, S. Chung, K. Cho, D. Harkin, W.-T. Hwang, D. Yoo, J.-K. Kim, W. Lee, Y. Song, H. Ahn, Y. Hong, H. Sirringhaus, K. Kang, T. Lee, *Adv. Mater.* **2019**, 31, 1806697.

[S30] K. Cho, J. Pak, J.-K. Kim, K. Kang, T.-Y. Kim, J. Shin, B. Y. Choi, S. Chung, T. Lee, *Adv. Mater.* **2018**, 30, 1705540.

[S31] X. Ma, *Adv. Mat. Res.* **2013**, 651, 193.

[S32] M. M. Furchi, D. K. Polyushkin, A. Pospischil, T. Mueller, *Nano Lett.* **2014**, 14, 6165.

[S33] J. Pak, I. Lee, K. Cho, J.-K. Kim, H. Jeong, W.-T. Hwang, G. H. Ahn, K. Kang, W. J. Yu, A. Javey, S. Chung, T. Lee, *ACS Nano* **2019**, 13, 9638.

[S34] J. Pak, M. Min, K. Cho, D.-H. Lien, G. H. Ahn, J. Jang, D. Yoo, S. Chung, A. Javey, T. Lee, *Appl. Phys. Lett.* **2016**, 109, 183502.

[S35] T.-Y. Kim, J. Ha, K. Cho, J. Pak, J. Seo, J. Park, J.-K. Kim, S. Chung, Y. Hong, T. Lee, *ACS Nano* **2017**, 11, 10273.

[S36] H. Xu, J. Wu, Q. Feng, N. Mao, C. Wang, J. Zhang, *Small* **2014**, 10, 2300.

[S37] C. Xie, C. Mak, X. Tao, F. Yan, *Adv. Funct. Mater.* **2017**, 27, 1603886.

[S38] H. Fang, W. Hu, *Adv. Sci.* **2017**, 4, 1700323.

[S39] L. Ju, J. Jr. Velasco, E. Huang, S. Kahn, C. Nosiglia, H.-Z. Tsai, W. Yang, T. Taniguchi, K. Watanabe, Y. Zhang, G. Zhang, M. Crommie, A. Zettl, F. Wang, *Nat. Nanotechnol.* **2014**, 9, 348.

[S40] M. Yankowitz, Q. Ma, P. Jarillo-Herrero, B. J. LeRoy, *Nat. Rev. Phys.* **2019**, 1, 112.
